# The neglected association between schizophrenia and bone fragility: a systematic review and meta-analyses

**DOI:** 10.1038/s41398-024-02884-1

**Published:** 2024-05-30

**Authors:** Behnaz Azimi Manavi, Kayla B. Corney, Mohammadreza Mohebbi, Shae E. Quirk, Amanda L. Stuart, Julie A. Pasco, Jason M. Hodge, Michael Berk, Lana J. Williams

**Affiliations:** 1 Deakin University, Institute for Mental and Physical Health and Clinical Translation-IMPACT, Geelong, VIC Australia; 2 Deakin University, Faculty of Health, Biostatistics unit, Geelong, Australia; 3 Institute of Clinical Medicine, Psychiatry, University of Eastern Finland, Kuopio, Finland; 4 Institute of Clinical Medicine, Kuopio Musculoskeletal Research Unit (KMRU), University of Eastern, Kuopio, Finland; 5 Barwon Health, Geelong, VIC Australia; 6 Department of Medicine-Western Health, The University of Melbourne, Parkville, VIC Australia; 7 Department of Epidemiology and Preventive Medicine, School of Public Health and Preventive Medicine, Monash University, Melbourne, VIC Australia; 8 Department of Psychiatry, The University of Melbourne, Parkville, VIC Australia; 9 Florey Institute of Neuroscience and Mental Health, Parkville, VIC Australia; 10 Orygen, The National Centre of Excellence in Youth Mental Health, Centre for Youth Mental Health, Parkville, VIC Australia

**Keywords:** Schizophrenia, Psychology

## Abstract

Schizophrenia is associated with increased risk of medical comorbidity, possibly including osteoporosis, which is a public health concern due to its significant social and health consequences. In this systematic review and meta-analysis, we aimed to determine whether schizophrenia is associated with bone fragility. The protocol for this review has been registered with PROSPERO (CRD42020171959). The research question and inclusion/exclusion criteria were developed and presented according to the PECO (Population, Exposure, Comparison, Outcome) framework. Schizophrenia was identified from medical records, DSM-IV/5 or the ICD. The outcomes for this review were bone fragility [i.e., bone mineral density (BMD), fracture, bone turnover markers, bone quality]. A search strategy was developed and implemented for the electronic databases. A narrative synthesis was undertaken for all included studies; the results from eligible studies reporting on BMD and fracture were pooled using a random effects model to complete a meta-analysis. The conduct of the review and reporting of results adhered to PRISMA guidelines. Our search yielded 3103 studies, of which 29 met the predetermined eligibility criteria. Thirty-seven reports from 29 studies constituted 17 studies investigating BMD, eight investigating fracture, three investigating bone quality and nine investigating bone turnover markers. The meta-analyses revealed that people with schizophrenia had lower BMD at the lumbar spine [standardised mean difference (SMD) −0.74, 95% CI −1.27, −0.20; Z = −2.71, p = 0.01] and at the femoral neck (SMD −0.78, 95% CI −1.03, −0.53; Z = −6.18, p ≤ 0.001). Also observed was a higher risk of fracture (OR 1.43, 95% CI 1.27, 1.61; Z = 5.88, p ≤ 0.001). Following adjustment for publication bias, the association between schizophrenia and femoral neck BMD (SMD −0.63, 95% CI −0.97, −0.29) and fracture (OR 1.32, 95% CI 1.28, 1.35) remained. Significantly increased risk of bone fragility was observed in people with schizophrenia. This association was independent of sex, participant number, methodological quality and year of publication.

## Introduction

Osteoporosis is a debilitating musculoskeletal disorder characterised by reduced bone stiffness, low bone mineral density (BMD), disruption of bone microarchitecture [1, 2] and increased fracture risk, particularly at the hip, spine and wrist [3–5]. It is a pressing public health concern due to related mortality, morbidity and disability [6–9]. Osteoporosis is often not detected until a fracture occurs; however, early detection is crucial to prevent subsequent fracture [10, 11]. Fracture can result in reduced quality of life and wellbeing, short-term morbidity, higher disability rate and related hospital admissions [8, 12], as well as heavy financial burden [13]. Thus, identifying subgroups of the population who may have an increased risk of bone fragility is imperative for prevention and anticipating health care needs.

Markers of bone integrity can be determined via several means. Dual energy x-ray absorptiometry (DXA) is a widely used technology that measures areal BMD to estimate fracture risk [4]. In addition, quantitative ultrasound (QUS) utilises sound waves to assess bone density and bone quality as measures of fracture risk, providing an alternative measure to DXA [14]. Recently, quantitative computed tomography (QCT) has been recognized for the diagnosis of osteoporosis. QCT is a three-dimensional technique for measuring BMD by assessing vertebrae from L1 to L4 [15]. Furthermore, bone turnover markers, by-products of the bone remodelling process, can also be examined as a contributor to fracture risk [10], however its clinical utility is limited due to the lack of data to date [16].

A number of medical conditions have been associated with bone health as well as some psychiatric disorders [17]. Increasing evidence is showing that people with schizophrenia have a higher prevalence of medical comorbidity [18], including osteoporosis and fracture [19–21]. Schizophrenia is a severe and chronic psychiatric disorder characterised by delusions, hallucinations and cognitive impairments, with approximately 1% of the general population meeting diagnostic criteria [22]. Many studies have shown that those with schizophrenia have reduced BMD and are more prone to developing osteoporosis and related fracture [19, 23–27], which could be due to certain lifestyle choices [28–30], antipsychotic-induced hyperprolactinemia [19, 31–33] or schizophrenia itself [20, 34]. Osteoporotic fracture is associated with several adverse outcomes in those living with schizophrenia including reduced well-being [35], higher rates of adverse events post fracture [36, 37], acute post-operative complications and longer hospital stays [38]. Given these consequences, a detailed and up to date synthesis of the existing evidence on the association between schizophrenia and bone fragility is therefore crucial.

Systematic reviews and meta-analyses have been conducted on this topic [19, 26, 39–41]. In the first systematic review of the literature, Kishimoto et al. [19] found that 15 of the 16 included studies showed osteoporosis was more prevalent among patients with schizophrenia compared to controls [19]. The next systematic review and meta-analysis included 19 studies—showing people with schizophrenia had significantly reduced BMD and a higher odds of osteoporosis compared to controls [39]. In another systematic review and meta-analysis by Stubbs et al. [26], individuals with schizophrenia had significantly lower bone mass compared to controls [26], which was similarly reported in a systematic review and meta-analysis by Tseng et al. [40] published in the same year [40]. Finally, Gomez et al. [41] investigated the BMD of patients with schizophrenia and reported significantly reduced BMD at their lumbar spine and hip [41].

In the current study, we aimed to build upon the early works in several ways: (I) comprehensively investigating associations between schizophrenia and bone fragility by examining bone quality and bone turnover markers as additional outcomes of interest, along with BMD and fracture risk; (II) examining associations between schizophrenia and bone fragility in both population and clinical settings; and (III) undertaking assessment of the methodological quality of the included studies, and adhering to relevant guidelines for the conduct of systematic review and meta-analyses [42].

Therefore, the aims of this review are to: (I) synthesise the existing evidence on associations between schizophrenia and bone fragility (defined as BMD, fracture, bone quality and bone turnover markers); (II) assess the quality of the included studies; and (III) explore sources of heterogeneity that might explain the observed findings.

The results that are derived from this study should provide an up-to-date and well-rounded synthesis of the bone health of people with schizophrenia. It is intended that this systematic review and meta-analyses will be relevant for informing clinical practice and policy as it will provide detailed information on the types and extent of evidence on associations between schizophrenia and bone health.

## Methods

The protocol [43] for this systematic review has been published and registered with the International Prospective Register of Systematic Reviews (PROSPERO, CRD42020171959). In addition, the Preferred Reporting Items for Systematic Reviews and Meta-Analyses (PRISMA) statement on systematic reviews was followed [42].

### Selection criteria

The research question and inclusion/ exclusion criteria were developed using a PECO structure (Population, Exposure, Comparison, Outcome):

- Population: Adult populations aged 18 years or older from clinical samples or the general population.
- Exposure: Schizophrenia recorded in medical records or identified using criteria from any version of the Diagnostic and Statistical Manual of Mental Disorders (DSM) [44] or the International Classification of Disease (ICD) [45].
- Comparison: Only studies with an appropriate comparison group, such as participants with no history of schizophrenia or other psychiatric disorders were eligible. Furthermore, case-control studies comparing the number of people with schizophrenia with and without a normal bone fragility outcome were included.
- Outcome: Bone fragility assessed by (I) BMD measures including T-score, Z-score, g/cm^2^, osteoporosis (defined as a BMD T-score of -2.5 or lower at the spine or femoral neck [46]) or osteopenia (defined as a BMD T-score of between -1 and -2.5 at the spine or femoral neck [46]) captured by any scanning machine including (DXA/ QUS/ QCT), (II) Bone quality was defined as any value measured by QUS machine [BUA: broadband ultrasound attenuation (dB/MHz)/ SI: stiffness index/ SOS: speed of sound (m/sec)], (III) Fracture was defined as any record of bone fracture at any skeletal site confirmed by ICD diagnosis, radiograph or medical practitioner. [4] Bone turnover markers were defined as any marker of bone resorption or bone formation. Bone turnover markers were grouped into bone resorption markers (including total pyridinoline (PYD), total deoxypyridinoline (DPD), C-terminal telopeptide of type I collagen (CTX/ICTP), TRAC-5b) and bone formation markers (alkaline phosphatase (ALP), bone alkaline phosphatase (BALP), osteocalcin (OC), P1CP, P1NP) [47].

Full-text published observational studies (cohort, cross-sectional and/or case-control) were eligible for inclusion in this study. Eligible studies were not restricted based on the sex or nationality of the sample, publication year or language.

### Exclusions

Articles were considered ineligible if they:

- were grey literature including theses
- were conference presentations/published abstracts
- had non-analytical epidemiological study designs such as clinical trials [baseline data from prospective studies (if available) were considered eligible] and case reports
- were review/systematic reviews
- had a population under the age of 18 years
- had a population with schizophrenia mixed with other psychiatric disorders
- did not examine schizophrenia according to inclusion criteria
- did not examine outcomes according to the inclusion criteria.

### Search strategy and information source

The search strategy to identify peer-reviewed literature was developed in consultation with an academic librarian (BK) and was informed by previous literature. It was developed for Medline Complete using index terms (e.g., MeSH) and keywords, Boolean Operators, relevant truncation and wildcard symbols, and explode functions. The search strategy was translated for CINAHL Complete, Embase, and PsycINFO. One reviewer implemented the search strategy and managed the records (BAM). The first search was conducted on 25 September 2020. In addition, the reference lists of prior systematic reviews and meta-analyses on this topic were hand-searched [19, 26, 39–41]. A secondary search was performed on the 12th of December 2022 to identify any new studies. Full details of the search strategy are presented in the supplementary online tables (Supplement Tables 1 and 2).

### Selection process

**Fig. 1:**
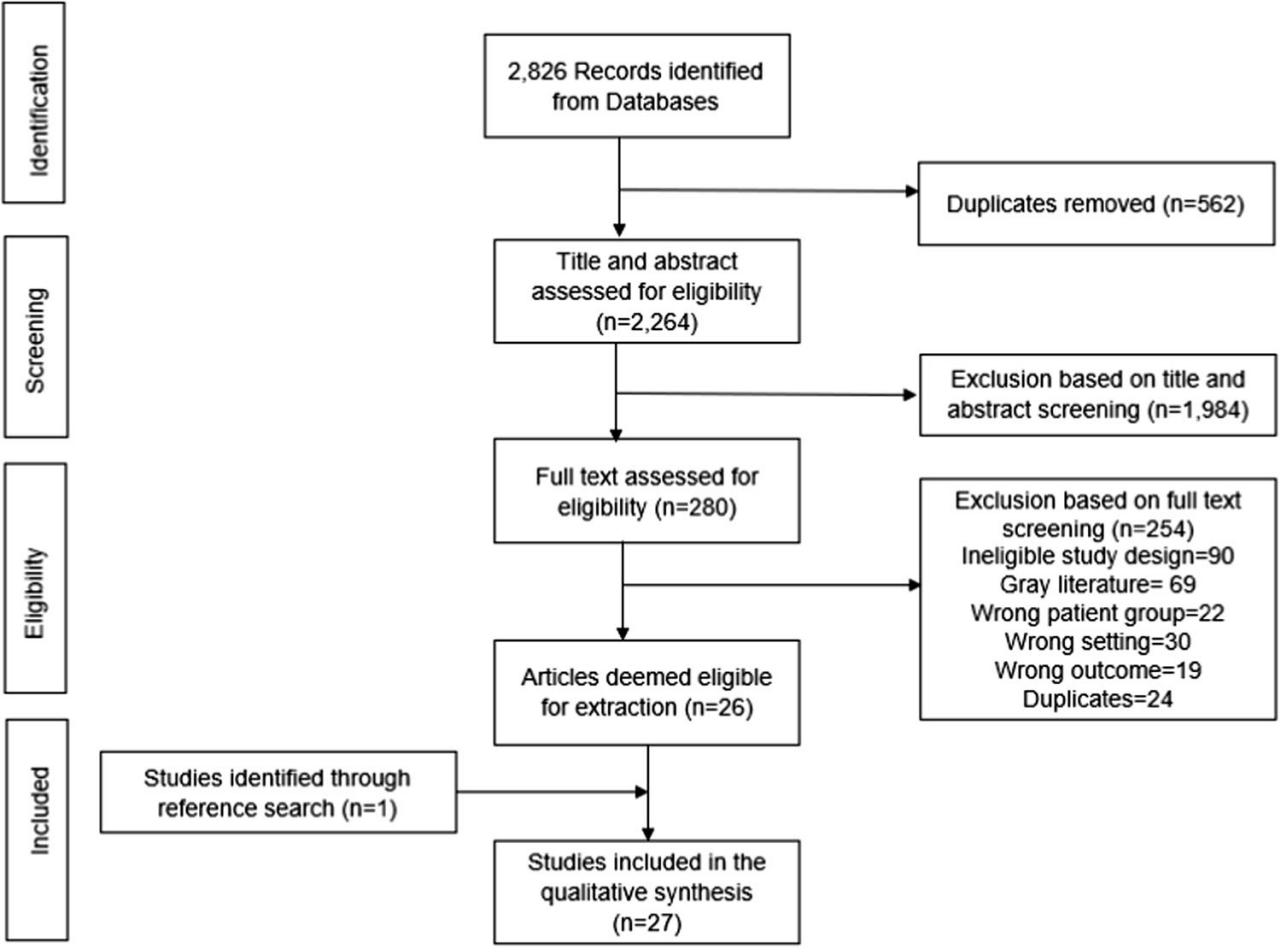
PRISMA flowchart 1. Flow diagram for studies included in this review from the first search.

**Fig. 2:**
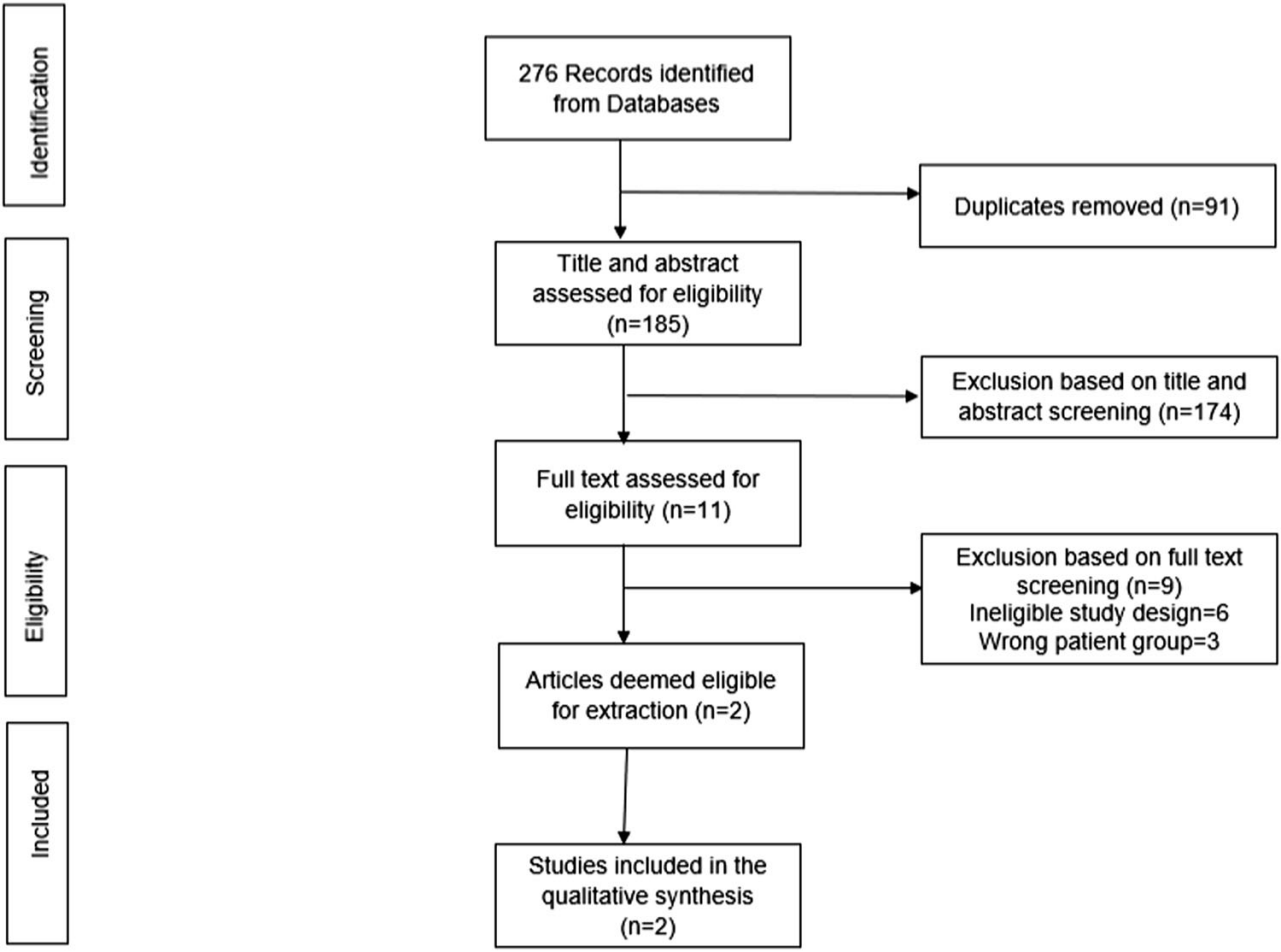
PRISMA flowchart 2. Flow diagram for studies included in this review from the second search.

Two reviewers (BAM and KBC) independently screened the titles/abstracts and full text of eligible articles in Covidence [48]. Where the two reviewers did not agree on decisions at the screening and/or the full-text stage, the supervisory reviewer (LJW) provided the final consensus. In addition, the supervising author performed a cross-check of 10% of the excluded articles. A flow diagram for studies included in this review and reasons for participation are shown in Figs. 1, 2.

As the current study was not limited to the English language, several studies published in Turkish, Polish and Chinese languages were identified. Google Translate was used for full text screening. After the full text screening, the Turkish [49] and Polish [50] studies were considered eligible and Google Translate was used for extracting the data.

### Data collection and extraction

A data extraction form was developed by two reviewers (BAM and SEQ) and data were extracted by one reviewer (BAM) under the guidance of a statistician (MM) and the supervisory author (LJW).

One reviewer (BAM) extracted the following parameters for each study: first author, publication year, geographical location, duration of data collection and data source (if applicable), study design, participant details (number, % female, menopause status, age), method of schizophrenia diagnosis, and bone fragility assessment details [device, site, values (DXA: T-score, Z-score, g/cm^2^, QUS: BUA, SI, SOS), ascertainment method for bone turnover markers, and the body site for fracture] and summary of the association between schizophrenia and bone fragility. If a study examined the relationship between more than one outcome of interest and schizophrenia, all relevant data were identified and extracted. Authors of eligible reports were contacted for data queries and/or requests.

Two independent reviewers (BAM and KBC) scored the methodological quality of included studies according to the National Institute of Health (NIH) [51]. NIH developed a methodological quality assessment tool for reviewers to focus on key concepts for the internal validity of studies such as assessment of study population, assessment of risk, analysis and data presentation, study design, and assessment of outcome [51]. We used the 14-item checklist for observational cohort and cross-sectional studies, and the 12-item checklist for case-control studies in this systematic review, respectively. Any discrepancies in the scoring were resolved through one census meeting with the supervisory reviewer (LJW).

### Narrative synthesis and statistical analyses

The reporting of results from this review are consistent with the PRISMA guidelines [42].

The results from all the included studies are presented in a qualitative synthesis including a description of the studies, with key findings presented in tables/text according to each outcome of interest.

Due to the dearth of available studies and heterogeneity (see Section “Heterogeneity and publication bias”) regarding the bone quality and bone turnover markers outcomes, only studies that examined two outcome variables (BMD and fracture) were selected for the meta-analyses. As potential heterogeneity was anticipated, all analyses were conducted in Stata 17 using a restricted maximum likelihood random-effects model (REML estimation method). Hedge’s g was considered the main effect size for the meta-analyses for continuous variable (BMD) with the odds ratio (OR) being considered the main effect size for the binary outcome (fracture-yes). Further information regarding the analyses is presented in the sections below (see Sections “BMD”–“Heterogeneity and publication bias”).

### BMD

Mean BMD values at each skeletal site for the schizophrenia and control groups were extracted. Standardised mean difference (SMD) and 95% Confidence Intervals (CI) were calculated and reported. Since studies varied in reporting the measured values of BMD and their sample sizes, we employed Hedges’ g effect size, which provides an unbiased result for studies with small sample sizes. The 95% CI of the effect size was also computed. Effect sizes were classified as small if ≤0.2, medium ≤04, or large ≥0.8 [52].

To reduce heterogeneity, only studies using a DXA machine as the ascertainment method for BMD were considered for the meta-analyses. As the lumbar spine and femoral neck were the most studied sites among the included studies, and have a better predictive ability of fracture [53], the studies with data on these two sites were considered for meta-analysis, with separate meta-analyses conducted for each site. We prioritised reporting BMD values in the following order: T-score, Z-score and absolute value, and consistent with the literature, we considered the T-score most relevant value for these analyses [54]. For prospective studies which grouped individuals with schizophrenia based on their medication use status at baseline; a formula [55] was used to convert the mean and standard deviation of these patients as a whole (irrespective of the medications). Data were analysed separately for males and females to reduce heterogeneity using the coherence guideline [55].

### Fracture

All recorded fracture sites, including hip, MOF, non-MOF, fracture rate, total fracture, and any fracture, were considered for meta-analysis. If studies reported hazard ratios (HR) and/or rate ratios (RR), these values were considered close estimates of OR [56] and the ORs were log-transformed for meta-analysis; however, for convenience, the final results were logged back-transformed and reported in the original metric.

If studies used data from different timepoints of the same source, the most recent study was considered for meta-analysis. As per the BMD outcome, males and females were analysed separately for the fracture outcome. If the number of males and females were not reported, we contacted the authors for this information. If we did not receive a response, a formula from Cochrane guidelines was used for analysing male and female information separately [55]. Moreover, if studies reported more than one fracture as the outcome, to not duplicate the number of samples and controls, the number of participants were divided based on the coherence guidelines [55].

### Heterogeneity and publication bias

Heterogeneity was assessed by calculating I^2^ and H^2^ values. An I^2^ score of 25% was considered as low, 50% as medium and 75% as high heterogeneity [55]. Heterogeneity was further explored via subgroup analyses, including sample size, year of publication, methodological quality, and site (for fracture only). Small-study effects were assessed using funnel plots and regression-based Egger [57] and Begg [58] tests. When publication bias was suspected, contour-enhanced funnel plots were generated, a trim-and-fill analysis [59] by Duval and Tweedie was performed and adjusted results with the trim-and-fill method were reported.

## Results

### Study selection

Figures 1 and 2 depict the results of the search strategy separately for each search, including the eligible and excluded studies at each stage of assessment. Overall, the e-search yielded 3102 articles, from which Covidence identified and removed 653 duplicates. A total of 2449 articles remained for title and abstract screening, with 2158 excluded. Following this, 291 full-text articles were assessed, of which 263 articles were excluded with reasons (96 had ineligible study design, 69 were grey literature, 25 wrong patient group, 30 wrong setting, 19 wrong outcome, and 24 further duplicates). The remaining 28 articles were considered for data extraction and quality assessment. A further article was found by hand-searching the reference lists of related systematic reviews. Thus, 29 articles were eligible for inclusion in this review.

### Methodological quality of included studies

The overall mean methodological quality score was 56.4% (range 23.3–83.3%), see Tables 1–4.

**Table 1:** Characteristics and summary of results of the included studies investigating schizophrenia and bone mineral density.

Citation (Country)	Study design, setting (years)	Exposure diagnostic criteria	Outcome assessment (type)	Subjects	Study population characteristics		BMD site assessed	Results			Matching Ratio (sample/case: control) Adjusting confounders	NIH score
					N=total Female N (% of total) Menopause status	Mean age ± SD (range)		Presented value	Mean ± SD/OR			
Altindaǧ et al. 2009 [49] (Turkey)	Nr Clinical (Jul-Dec 2008)	DSM-IV	DXA (Hologic QDR 4500)	SCHz	N = 30 F (100%) Premenopausal	42.8 ± 5.3 (23-52)	LS	T-score g/cm^2^	−0.6 0.59	p<0.001 p<0.001 p<0.001 p<0.001	Matched for: sex Ratio: Nr Adjusted for: Na	4/13
							FN	T-score g/cm^2^	−0.8 0.47			
				HC	N = 40 F (100%) Nr	36.9 ± 8.4 (32-51)	LS	T-score g/cm^2^	0.9 1.04			
							FN	T-score g/cm^2^	1.3 1.07			
Bergemann et al. 2008 [60] (Germany)	Nr Clinical (Nr)	DSM-IV ICD-10	DXA (Lunar-DPXL device (Lunar Corporation, Madison, WI, USA)	SCHz	N = 72 F (100%) Premenopausal	33.8 ± 6.5 (20.5-45.3)	LS (L1-L4)	T-score g/cm^2^	−0.11 ± 1.5 1.186	p = 0.493	Matched for: age, sex, ethnic Ratio: Nr Adjusted for: Na	5/13
							FN	T-score g/cm^2^	−0.22 ± 1.0 0.953	p = 0.121		
				HC	N =71 F (100%) Nr	33.7 ± 6.3 (20.9-45.9)			Nr			
Bolton et al. 2011 [61] (Canada)	Case-control Population-based (2000-2007)	ICD-9-CM ICD-10-CA	DXA (Lunar Prodigy; GE Lunar, Madison, Wis)	SCHz	low BMD = 30 (0.4) F (100%) Postmenopausal Normal BMD = 38 (0.2) F (100%) Postmenopausal ₳	Nr	LS (L1-L4), FN, Trochanter, Hip	T-score	AOR = 1.98; (95% CI, 1.04, 3.77) p<0.05		Matched for: age, sex, ethnicity R: 1:3 Adjusted for: socioeconomic status, BMI, medical comorbidity, heart disease, diabetes, hypertension, COPD, renal disease, cirrhosis, thyroid disease, inflammatory bowel disease, celiac disease, individual mental disorders, systemic estrogens, raloxifene, calcitonin, bisphosphonates, corticosteroids, antiandrogens, antiestrogens, thiazide diuretics, individual psychotropic categories	8/12
Chen et al. 2021 [74] (China)	Cross-sectional Clinical (May 2018-June 2020)	DSM-IV	QUS (Sahara clinical bone sonometer, Hologic)	SCHz	N = 250 F 113 (45.20%) Nr	46.3 ± 10.6 (18-75)	Calcaneus (right heel)		Osteoporosis 24.4% (61/250) P <0.001		Similar age, sex	7/13
				HC	N = 288 F 151 (52.43%) Nr	46.0 ± 10.8			Osteoporosis 10.1% (29/288)			
Chiang et al. 2020 [62] (Taiwan)	Cross-sectional Clinical	DSM-IV	DXA (Nr)	SCHz	N = 47 Out/inpatients F 20 (42.55%) Nr	41.9 ± 10.04 (Nr)	LS	T-score Z-score g/cm^2^	−0.0021 ± 1.38 0.34 ± 1.11 1.03 ± 0.16	p = 0.843 p = 0.731 p = 0.821	Unmatched Ratio: Nr Adjusted for: Na	7/13
				HC	N = 39 F 28 (71.79%) Nr	39.4 ± 8.1 (18-65)		T-score Z-score g/cm^2^	0.05 ± 1.26 0.28 ± 1.13 1.02 ± 0.15			
Cui et al. 2017 [63] (China)	Cross-sectional Clinical (Jun 2014-Dec 2015)	SCID	QUS (3.01 Sahara Clinical Bone Sonometer Hologic) T-score	SCHz	N = 199 F 67(33.7%) Nr	54.5 ± 11.1 (18-83)	Calcaneus (right heel)		−1.11 ± 1.76 0.75 ± 1.96 (M) −1.77 ± 1.11 (F)	p<0.05	Unmatched Ratio: Nr Adjusted for: sex, age, education, BMI, smoking	7/13
				HC	N = 107 F 85 (76.7%) Nr	41.7 ± 11.9 (18-75)			−0.67 ± 1.38			
Doknic et al. 2011 [64] (Serbia)	Prospective cross-sectional study Clinical (Nr)	DSM-IV	DXA (Discovery; Hologic Inc., Waltham, Mass., USA)	SCHz	N = 26 F 14 (53.84%) Nr	31.3 ± 1.3 (Nr)	LS	g/cm^2^ Z-score	1.01 ± 0.03 −0.38 ± 0.2	p = 0.094 p = 0.072	Matched for: age, sex, BMI, Education Ratio: Nr Adjusted for: Na	5/13
							FN	g/cm^2^ Z-score	1.00 ± 0.03 0.29 ± 0.22	p = 0.560 p = 0.386		
				HC	N = 35 F 24 (68.57%) Nr	32.2 ± 1.4 (Nr)	LS	g/cm^2^ Z-score	1.07 ± 0.02 0.23 ± 0.2			
							FN	g/cm^2^ Z-score	1.02 ± 0.02 0.52 ± 0.17			
Du et al. 2020 [65] (China)	Nr Clinical (Nr)	DSM-5 SCID-P	QCT (Toshiba Aquilion 64-row CT scanner)	SCHz	N = 79 M 100% Na	Mean age = 46 (36-57)	L2–L4 vertebral cancellous	Mg/cm^3^	IQR = 88.7–130.4	p = 0.001	Matched for: sex Ratio: Nr Adjusted for: Na	6/13
				HC	N = 56 M 100% Na	Mean age = 44 (Nr)			IQR = 113.1–146.03			
Jung et al. 2006 [66] (Korea)	Cross-sectional Clinical (Jan 2005-Jun 2005)	DSM-IV	DXA (GE Lunar 4500 scanner)	SCHz	N = 51 inpatients F 21 (41.17%) Nr	40.4 ± 4.7 (18-45) F = 37.8 ± 5.5 (Nr) M = 39.9 ± 5.1 (Nr)	LS (L1–L4)	g/cm^2^	1.13 ± 0.13 1.09 ± 0.13 (F) 1.16 ± 0.13 (M)	p<0.05 p<0.005 Ns	Unmatched Ratio: Nr Adjusted for: Na	6/13
							FN		0.89 ± 0.14 0.80 ± 0.11 (F) 0.95 ± 0.14 (M)	p<0.005 p<0.005 Ns		
							WARD		0.78 ± 0.14 0.71 ± 0.13 (F) 0.84 ± 0.13 (M)	p<0.005 p<0.005 Ns		
							TROCH		0.77 ± 0.14 0.68 ± 0.09 (F) 0.83 ± 0.12 (M)	p<0.005 p<0.005 Ns		
							LS	T-score	−0.14 ± 1.06 −0.13 ± 1.08 (F) −0.15 ± 1.07 (M)	p<0.05 p<0.005 Ns		
							FN		−0.35 ± 1.07 −0.81 ± 0.91 (F) −0.02 ± 1.06 (M)	p<0.005 p<0.005 Ns		
							WARD		−0.73 ± 1.11 −1.29 ± 1.01 (F) −0.34 ± 1.02 (M)	p<0.005 p<0.005 Ns		
							TROCH		0.00 ± 1.16 −0.67 ± 0.84 (F) 0.46 ± 1.14 (M)	p<0.005 p<0.005 Ns		
				HC	N = 57 F 23 (40.35%) Nr	Mean age = 38.3 (similar to SCHz group) F = 40.3 ± 3.3 (Nr) M = 36.9 ± 6.5 (Nr)	LS	g/cm^2^	1.20 ± 0.13 1.20 ± 0.12 (F) 1.20 ± 0.13 (M)			
							FN		0.97 ± 0.13 0.93 ± 0.14 (F) 1.00 ± 0.11 (M)			
							WARD		0.87 ± 0.15 0.87 ± 0.18 (F) 0.87 ± 0.12 (M)			
							TROCH		0.85 ± 0.13 0.80 ± 0.12 (F) 0.88 ± 0.13 (M)			
							LS	T-score	0.39 ± 1.11 0.74 ± 1.01 (F) 0.16 ± 1.13 (M)			
							FN		0.32 ± 0.99 0.21 ± 1.15 (F) 0.40 ± 0.87 (M)			
							WARD		−0.13 ± 1.13 −0.17 ± 1.38 (F) −0.11 ± 0.95 (M)			
							TROCH		0.73 ± 1.19 0.46 ± 1.12 (F) 0.91 ± 1.22 (M)			
Jung et al. 2011 [67] (Korea)	Cross-sectional Clinical (Nr)	DSM-IV	Radiograph, medical reports (Na)	SCHz	N = 229 F 93 (40.6%) Postmenopausal	58.7 ± 6.8 (>50) F= 59.1 ± 7.8 (>50) M = 58.2 ± 5.7 (>50)	LS (L1–L4)	g/cm^2^	1.05 ± 0.20 0.98 ± 0.20 (F) 1.10 ± 0.18 (M)	p<0.05 p<0.005 Ns	Unmatched Ratio: Nr Adjusted for: Na	7/13
							FN		0.82 ± 0.16 0.77 ± 0.17 (F) 0.85 ± 0.14 (M)	p<0.005 p<0.005 p<0.005		
							WARD		0.69 ± 0.18 0.65 ± 0.20 (F) 0.71 ± 0.16 (M)	p<0.05 Ns p<0.05		
							TROCH		0.74 ± 0.14 0.68 ± 0.15 (F) 0.77 ± 0.13 (M)	p<0.005 p<0.005 p<0.005		
							LS	T-score	−0.82 ± 1.68 −0.03 ± 1.90 (F) −0.68 ± 1.51 (M)	p<0.005 p<0.005 Ns		
							FN		−0.85 ± 1.22 −1.02 ± 1.40 (F) −0.73 ± 1.06 (M)	p<0.005 p<0.005 p<0.005		
							WARD		−1.47 ± 1.38 −1.76 ± 1.51 (F) −1.28 ± 1.25 (M)	p<0.05 Ns p<0.005		
							TROCH		−0.29 ± 1.28 −0.60 ± 1.39 (F) −0.08 ± 1.17 (M)	p<0.005 p<0.005 p<0.005		
				HC	N = 125 F 60 (48%) postmenopausal	58.6 ± 6.4 (similar to SCHz group) F = 58.2 ± 6.0 (Nr) M = 59.0 ± 6.8 (Nr)	LS	g/cm^2^	1.11 ± 0.18 1.06 ± 0.13 (F) 1.16 ± 0.22 (M)			
							FN		0.92 ± 0.15 0.89 ± 0.11 (F) 0.94 ± 0.18 (M)			
							WARD		0.73 ± 0.14 0.70 ± 0.13 (F) 0.76 ± 0.14 (M)			
							TROCH		0.80 ± 0.14 0.75 ± 0.10 (F) 0.85 ± 0.15 (M)			
							LS	T-score	−0.33 ± 1.36 −0.41 ± 1.08 (F) −0.26 ± 1.58 (M)			
							FN		−0.22 ± 0.98 −0.10 ± 0.92 (F) 0.05 ± 1.04 (M)			
							WARD		−1.14 ± 1.09 −1.39 ± 1.01 (F) −0.91 ± 1.11 (M)			
							TROCH		0.42 ± 1.06 0.03 ± 0.87 (F) Nr			
Keely et al. 1997 [68] (Canada)	Cross-sectional Clinical (Nr)	Nr	DXA (DPX, software version 3.4; Lunar Corp., Madison, WI)	SCHz	N = 16 M 100% Na	41.3 ± 2.9 (19-62)	LS FN WARD TROCH	g/cm^2^	1.107 ± 0.039 0.980 ± 0.041 0.854 ± 0.053 0.877 ± 0.041	p<0.001 p=0.090 p=0.046 p=0.021	Matched for: age, sex, BMI Ratio: Nr Adjusted for: Na	4/13
				HC	N = 16 M 100% Na	41.0 ± 3.1 (Nr)	LS FN WARD TROCH		1.285 ± 0.031 1.079 ± 0.039 0.998 ± 0.031 1.001 ± 0.031			
Kishimoto et al. 2008 [69] (Japan)	Nr Clinical (Feb - May 2005)	DSM-IV	DXA (Aloka Dicroma scan DCS-600EX-3, Aloka Co., Ltd., Tokyo, Japan)	SCHz	N = 74 M 100% Na	58.9 ± 12.2 (31-78)	Distal radius contralateral		Osteopenia 37.8% (28/74), osteoporosis 27.0% (20/74) p<0.05 in all but 3 of the 10 age groups (30–34, 35–39, 50–54years)		Matched for: age, sex, race Ratio: Na Adjusted for: Na	6/13
				HC	Reference data	Nr						
Koçer et al. 2011 [70] (Turkey)	Cross-sectional Clinical (nr)	DSM IV	DXA (Ge-Lunar Dpx Nt Pro Lunar Corp, Adison, WI, USA)	SCHz	N = 14 F 5 (35.71%%) Nr	33.07 ± 8.92 (24-42)	LS (L1-L4) FN	T-score	−0.60 ± 1.40 −0.66 ± 0.99	P=0.04 P=0.02	Similar age, sex	5/13
				HC	N = 31 F 16 (51.61%) Nr	34.3 ± 7.0 (22-44)	LS (L1-L4) FN		0.20 ± 1.15 0.16 ± 1.08			
Liang et al. 2019 [71] (China)	Nr Clinical (Jan 2015- Jan 2016)	DSM-IV	DXA (Discovery WI DXA Hologic, USA) g/cm^2^	SCHz	N = 150 F 83 (55.33%) Nr	25.7 ± 4.9 (18-40)	L1	g/cm^2^	0.87 ± 0.09 (olanzapine) 0.84 ± 0.12 (drug-naïve)		Matched for: age, sex Ratio: Nr Adjusted for: Na	5/13
							L2		0.93 ± 0.11 (olanzapine) 0.90 ± 0.12 (drug-naïve)			
							L3		0.95 ± 0.12 (olanzapine) 0.93 ± 0.11 (drug-naïve)			
							L4		0.95 ± 0.12 (olanzapine) 0.91 ± 0.11 (drug-naïve)			
							LS		0.94 ± 0.12 (olanzapine) 0.89 ± 0.12 (drug-naïve)			
							FN		0.79 ± 0.11 (olanzapine) 0.76 ± 0.13 (drug-naïve)			
							TROCH		0.65 ± 0.09 (olanzapine) 0.62 ± 0.08 (drug-naïve)			
							WARD		0.74 ± 0.13 (olanzapine) 0.73 ± 0.14 (drug-naïve)			
							T-Hip		0.88 ± 0.13 (olanzapine) 0.84 ± 0.11 (drug-naïve)			
							1/3: radius ulna		0.68 ± 0.07 (olanzapine) 0.67 ± 0.08 (drug-naïve)			
							MID: radius ulna		0.56 ± 0.07 (olanzapine) 0.55 ± 0.07 (drug-naïve)			
							UD: radius ulna		0.43 ± 0.13 (olanzapine) 0.41 ± 0.06 (drug-naïve)			
							T: radius ulna		0.55 ± 0.07 (olanzapine) 0.54 ± 0.06 (drug-naïve)			
				HC	N = 71 F 40 (56.34%) Nr	25.9 ± 4.7 (age matched)	L1		0.89 ± 0.08	p=0.023		
							L2		0.96 ± 0.08	p=0.018		
							L3		0.99 ± 0.08	p=0.005		
							L4		1.01 ± 0.08	p<0.001		
							LS		0.97 ± 0.07	p=0.002		
							FN		0.82 ± 0.07	p=0.003		
							TROCH		0.69 ± 0.07	p<0.001		
							WARD		0.78 ± 0.08	p=0.066		
							T-Hip		0.91 ± 0.09	p=0.017		
							1/3:radius ulna		0.72 ± 0.05	p=0.275		
							MID:radius ulna		0.64 ± 0.08	p<0.001		
							UD:radius ulna		0.47 ± 0.03	p<0.001		
							T:radius ulna		0.64 ± 0.09	p<0.001		
Lin et al. 2019 [72] (Taiwan)	Prospective Longitudinal Clinical (Nr)	DSM-IV	DXA (Nr)	SCHz	N =111 F 46 (41.44%) Nr	Mean age = 41.5 (Nr)	LS	T-score	−0.6 ± 1.1 (non-clozapine) 0.2 ± 1.5 (clozapine)		Matched for: age Ratio: Nr Adjusted for: Na	8/13
								Z-score	−0.3 ± 1.1 (non-Clozapine) 0.4 ± 1.5 (clozapine)			
				HC	N = 44 F 31 (70.5%) Nr	40.2 ± 8.6 (Nr)		T-score Z-score	0.0 ± 1.2 0.3 ± 1.1	p=0.003 p=0.008		
Wang et al. 2014 [73] (China)	Prospective cohort Clinical (May 2010-Apr 2012)	ICD-10 revision	DXA (American General Medical Equipment Co)	SCHz	N = 163 F 77 (47.2%) Nr	Mean age = 34.5 (25-45)	L1	g/cm^2^	1.08 ± 0.16 (conventional)	p=0.09	Matched for: age, sex, BMI, marital status, years of education Ratio: Nr Adjusted for: Na	8/14
									1.04 ± 0.16 (atypical)	p=0.12		
							L2		1.19 ± 0.18 (conventional)	p=0.06		
									1.12 ± 0.17 (atypical)	p=0.14		
							L3		1.25 ± 0.17 (conventional)	p=0.17		
									1.14 ± 0.17 (atypical)	p=0.35		
							L4		1.26 ± 0.19 (conventional)	p=0.39		
									1.14 ± 0.15 (atypical)	p=0.08		
				HC	N = 90 F 42 (46.66%) Nr	34.2 ± 10.6 (Matched for age)	L1 L2 L3 L4	1.16 ± 0.12 1.24 ± 0.12 1.42 ± 1.23 1.28 ± 0.15				
Wyszogrodzka-Kucharska et al. 2005 [50] (Poland)	Nr Clinical (Nr)	Nr	DXA (Lunar apparatus, model prodigy, SN64159 by GE Medical system)	SCHz	N = 60 F 36 (60%) Premenopausal	31.1 ± 8.6 (20-50)	L2-L4	g/cm^2^	1.164 ± 0.15 1.170 ± 0.153 (F) 1.153 ± 0.149 (M)	p<0.05 p<0.05 Ns	Unmatched Ratio: Nr Adjusted for: Na	3/13
								Z-score	−0.52 ± 1.19 0.34 ± 1.14 (F) −0.81 ± 1.24 (M)	p<0.01 p<0.01 Ns		
				HC	N = 38 F 21 (55.26%) Nr	31.7 ± 8.0 (20-49)		g/cm^2^	1.237 ± 0.126 1.247 ± 0.112 (F) 1.226 ± 0.144 (M)			
								Z-score	0.19 ± 0.99 0.47 ± 0.80 (F) −0.14 ± 1.12 (M)			

*SCHz* Schizophrenia, *OR* Odds ratio, *SD* Standard Division, *CI* Confidence Interval, *SCID-I* Research Version of the Structured Clinical Interview for Diagnostic and Statistical Manual of Mental Disorders, *MOF* Major Osteoporotic Fracture, *DSM* Diagnostic and Statistical Manual of Mental Disorders, *ICD* International Classification of Diseases, *GPRD* General Practice Research Database, *DPCRR* Danish Psychiatric Central Research Register, *NHDS* National Hospital Discharge Survey, *NHIRD* National Health Insurance, *DXA* Dual energy x-ray absorptiometry, *QCT* Quantitative Computed Tomography, *QUS* Quantitative Ultrasound, *HC* Healthy control, *F* Female, *M* Male, *LS* Lumbar spine, *FN* Femoral neck, *Troch* Trochanter, *T-Hip* Total hip, *Nr* not reported, *Ns* Not significant; *Na* not applicable.

₳ it is a case control study; controls are schizophrenia with normal BMD.

**Table 2:** Characteristics and summary of the included studies investigating schizophrenia and fracture.

Citation (Country)	Study design, setting (years) Data source	Exposure diagnostic criteria	Outcome assessments	Fracture site	Characteristic of schizophrenia group		Characteristic of comparison group		Matching Ratio (sample/case: control) Adjusting confounders	Results N (%) OR (95% CI)	NIH score
					N = total Female N (% of total)	Mean age ± SD (range)	N = total Female N (% of total)	Mean age ± SD (range)			
Bishop et al. [79] (USA)	Retrospective controlled study Clinical (nr) Database of medical centre	ICD	Bone fracture recorded from electronic medical records	Nr	N = 46 F 46 (100%)	61.0 ± 12.2 (45–90)	N = 46 F (100%)	61.1 ± 11.9 (47–83)	Matched for: age, sex, frequency Ratio: 1:1	fractures in SCHZ G 6/46 (13%) fractures in HC G 1/46 (2%) P = 0.11 Total fracture in SCHZ 12/46 (26%) Total fracture in HC 1/46 (2%) P = 0.001	7/12
Bolton et al. [77] (Canada)	Case-control Population-based (Apr 1996-Mar 2004) Administrative health data from Manitoba Department of Health	DSM-IV CIE-10	ICD-9-CM	Vertebral Wrist Hip	SCHz with fracture: 142 (0.9) F Nr Nr	Nr	SCHz without fracture: 197 (0.4) ₳ F Nr Nr	Nr	Matched for: year of birth, sex, ethnicity, comorbidity index Ratio: 1:3 Adjusted for: demographic variables, medications (include antipsychotic), physical conditions, and mental disorders	SCHz in fracture group = 142/15,792 (0.9) SCHz in control group= 197/47,289 (0.4) OR (95% CI) = 2.17 (1.75, 2.69) FAO of osteoporotic fracture for SCHz= (OR = 1.61; 95% CI, 1.27, 2.04; P < 0.01)	10/12
Bolton et al. [78] (Canada)	Longitudinal Cohort Population-based (Jan1996-Mar 2013) Manitoba Bone Density Program database	ICD-9-CM ICD-10-CA	An incidence hip fracture or MOF ICD-9-CM ICD-10-CA	MOF: Hip Vertebral (clinical) Humerus Forearm	SCHz with MOF fracture: 19 (0.3) F Nr Nr	Nr	SCHz without MOF fracture: 172 (0.3) F Nr Nr	Nr	Matched for: Na Ratio: Nr Adjusted for: Model 1: FRAX score and prior use of osteoporosis medications. Model 2: adjusted for antipsychotic as well	Model 1: SCHz for incident fracture of MOF (aHR = 1.82; 95% CI, 1.16, 2.85; p < 0.05) SCHz for incident fracture of hip (aHR 2.34; 95% CI, 1.05, 5.21; p < 0.05)	8/14
										Model 2: SCHz for incident fracture of MOF (aHR 1.21; 95% CI, 0.75, 1.97) SCHz for incident fracture of hip (aHR 1.12, 95% CI, 0.48, 2.63)	
					SCHz with hip fracture: 6 (0.4) F Nr Nr		SCHz without hip fracture: 185 (0.3) F Nr Nr			MOF for SCHz (19 out of 191) vs. for non-mental disorders 4562 out of 55748 (p = 0.003) Hip for SCHz 6 out of 191 vs. for non-mental 1271 out of 55748 (p = 0.56)	
Chu et al. [81] (Taiwan)	Retrospective cohort Population-based (Nr) Longitudinal Health Insurance Database 2005 (LHID 2005)	ICD-9-CM	ICD-9-CM	Vertebral Wrist Hip	N = 2028 F 1002 (49.41%)	Nr	N = 8112 F 4008 (49.41%)	Nr	Matched for: age, sex Ratio: 1:4 Adjusted for: age, sex, comorbidities, degree of urbanization, income level	New diagnosed fracture for SCHz [89 out of 2028 (4.39%)] vs. for without SCHz [257 out of 8112 (3.17%)] (p < 0.01)	8/14
										Vertebral fracture for SCHz [53 out of 2028 (2.61%)] vs. for without SCHz [142 out of 8112 (1.75%)] (p = 0.01)	
										Wrist fracture for SCHz [18 out of 2028 (0.89%)] vs. for without SCHz [76 out of 8112 (0.94%)] (p = 0.84)	
										Hip fracture for SCHz [25 out of 2028 (1.23%)] vs. for without SCHz [54 out of 8112 (0.67%)] (p = 0.01)	
										Vertebral fracture: Univariate analysis: HR for schizophrenia (HR = 1.53; 95% CI 1.12, 2.10; p = 0.01) Multivariate analysis: HR for schizophrenia (HR = 1.40; 95% CI 1.01, 1.95; p = 0.05)	
										Wrist fracture: Univariate analysis: HR for schizophrenia (HR = 0.96; 95% CI 0.58, 1.61; p = 0.89) Multivariate analysis: HR for schizophrenia (HR = 0.96; 95% CI 0.57, 1.61; p = 0.88)	
										Hip fracture: Univariate analysis: HR for schizophrenia (HR = 1.89; 95% CI 1.18, 3.04; p = 0.01) Multivariate analysis: HR for schizophrenia (HR = 1.78; 95% CI 1.08, 2.93; p = 0.02)	
										Comparing risk of fracture in SCHz by sex: Vertebral fracture: Adjusted HR of schizophrenia for M (AHR = 1.62; 95% CI 0.91, 1.04), for F (AHR = 1.29; 95% CI 0.86, 1.94) (p = 0.55)	
										Wrist fracture: Adjusted HR of schizophrenia for M (AHR = 0.65; 95% CI 0.26, 1.62), for F (AHR = 1.25; 95% CI 0.65, 2.43) (p = 0.64)	
										Hip fracture: Adjusted HR of schizophrenia for M (AHR = 1.27; 95% CI 0.60, 2.65), for F (AHR = 2.68; 95% CI 1.32, 5.44) (p = 0.20)	
Howard et al. [76] (UK)	Case-control Population-based (Aug 1987-Nov 1999) GPRD	ICD-10	ICD-9 Hospital records of hip fracture & GP’s confirmation	FN Hip	SCHz with fracture=100 (0.61) F Nr Nr		SCHz without fracture=110 (0.37) ₳ F Nr Nr		Matched for: age, sex, general practice Ratio: 1:2 Adjusted for: sex, age, BMI, smoking, medical condition, antipsychotics	SCHz in fracture cases 100/16341 (0.61%) SCHz in controls without fracture 110/29889 (0.37%)	8/12
										Univariate analysis: OR of hip fracture in schizophrenia (OR = 1.73; 95% CI 1.32, 2.28)	
										F: AOR for hip fracture with schizophrenia (AOR = 1.01; 95% CI 0.72, 1.40; p = 0.971)	
										M: AOR for hip fracture with schizophrenia (AOR = 1.61; 95% CI 0.81, 3.19; p = 0.174)	
Jung et al. [67] (Korea)	Cross-sectional Clinical (Nr)	DSM-IV	Radiograph, medical reports	Any fracture	N = 229 F 93 (40.6%) Postmenopausal	58.7 ± 6.8 ( > 50) F = 59.1 ± 7.8 ( > 50) M = 58.2 ± 5.7 ( > 50)	N = 125 F 60 (48%) postmenopausal	58.6 ± 6.4 (similar to SCHz group) F = 58.2 ± 6.0 (Nr) M = 59.0 ± 6.8 (Nr)	Matched for: age, sex Ratio: Nr Adjusted for: Na	One fracture SCHz G: F 19 (20.4%), M 20 (14.7%) Two fractures SCHz G: F 5 (5.4%), M 5 (3.7%) Three or more fractures SCHz G: F 0 (0.0%), M 6 (4.4%) Total: F 24 (25.8%), M 31 (22.8%)	7/13
										One fracture HC G: F 4 (6.7%), M 3 (4.6%) Two fractures HC G: F 0 (0.0%), M 0 (0.0%) Three or more fractures HC G: F 0 (0.0%), M 0 (0.0%) Total: F 4 (6.7%), M 3 (4.6%)	
										Schizophrenia vs controls: (24.0% vs 5.6%; X^2^_1_ = 15.1572, p = 0.001)	
Sørensen et al. [80] (Denmark)	Nr Population-based (Jan 1995-Dec 2009) DPCRR	ICD-10 ICD-8	ICD-10	Hip	N = 15,431 F 6326 (41.0%) Nr	49.9 ± 13.4 (Nr)	N = 3,807,597 F 2,167,977 (57.0%) Nr	46.9 ± 17.5 (Nr)	Matched for: Na Ratio: Nr Adjusted for: Model 1: sex, age, education, early retirement pension, CPDS, lifetime DDD’s of corticosteroids, lifetime alcohol-related diagnosis, antidepressants, anticholinergics, and benzodiazepines Model 2: Model1 + antipsychotics	Model1: (IRR = 1.19, 95% CI 1.08, 1.31; p < .001) IRR of hip fracture for SCHZ G	7/12
										Model2: (IRR = 1.0, 95% CI 0.90, 1.11; p = 0.995)	
Tsai et al. [27] (Taiwan)	Retrospective cohort Population-based (1999-2000 FU=Jan2001-Dec 2010) NHIRD	ICD-9-CM	ICD-9	MOF: Spine Hip Humerus Forearm Wrist	N = 30,335 F 15,208 (50.13%) Nr	51.1 ± 9.8 (Nr)	N = 121,340 F 60,832 (50.13%) Nr Non-schizophrenic	51.1 ± 9.8 (Nr)	Matched for: age, sex Ratio: 1:4 Adjusted for: demographic data, osteoporotic fracture-related illness	MOF SCHZ = 1667 (5.53%) MOF HC = 4257 (3.51%) p < 0.0001 Non-MOF SCHZ = 1228 (4.05%) Non-MOF HC = 4886 (4.03%) p = 0.8652 OR = NR	10/14

*SCHz* Schizophrenia, *HC* healthy control, *OR* odds ratio, *SD* standard division, *CI* confidence interval, *DSM* Diagnostic and Statistical Manual of Mental Disorders, *ICD* International Classification of Diseases, *MOF* Major Osteoporotic Fracture, *DSM* Diagnostic and Statistical Manual of Mental Disorders, *GPRD* General Practice Research Database, *DPCRR* Danish Psychiatric Central Research Register, *NHDS* National Hospital Discharge Survey, *NHIRD* National Health Insurance Research Database, *M* Male, *F* female, *Nr* not reported, *Ns* not significant, *FU* follow up, *FAO* fully adjusted odds ratio, *FN* femoral neck.

₳ it is a case control study, control group is SCHz without fracture.

**Table 3:** Characteristics and summary of the included studies investigating schizophrenia and bone quality.

Citation (Country)	Study design, setting (years) Data source	Exposure diagnosis	Outcome assessments	QUS value assessed	Study population with schizophrenia		Study population without schizophrenia		Matching Ratio (sample/case: control) Adjusting confounders	Results Mean (95% CI)	NIH score
					N = total Female N (% of total) Menopause status	Mean age ± SD (range)	N = total Female N (% of total) Menopause status	Mean age ± SD (range)			
Partti et al. [34] (Finland)	Cross-sectional Population-based (Sep 2000 -Jun 2001) Health 2000 Survey	SCID-I/medical records	QUS: Sahara Clinical Bone Sonometer (Hologic, Inc., Bedford, Massachusetts)	BUA(dB/MHz) SOS(m/sec) Z-score	N = 48 F 28 (53.33%) (71.7-non-menstruating)	Mean age = 53.5 (Nr)	N = 6100 F 3355 (55%) Nr	Nr	Matched for: age, sex Ratio: Nr Adjusted for: Model 1: BMI Model 2 (M): Model1 + low calcium diet, vitamin D, smoking, grip strength, education, alcohol consumption Model 2 (F): Model1 + low calcium diet, vitamin D, smoking, grip strength, oral estrogen preparations, menstruation status Model 3 (M): Model 2 (M) + antipsychotic medication and mood stabilizing medication Model 3 (F): Model 2 (F) + antipsychotic medication and mood stabilizing medication	BUA = 70.4 (64.7 to 76.1) (SCHz) SOS = 1534.3 (1524.8 to 1543.8) (SCHz) Z-BUA = −0.29 (−0.82 to 0.24) (M, SCHz) Z-SOS = −0.49 (−1.03 to 0.04) (M, SCHz) Z-BUA = −0.54 (−0.88 to −0.21) (F, SCHz) Z-SOS −0.53 (−0.87 to −0.19) (F, SCHz)	7/13
										Model1 (F): Z-BUA −0.65(−0.98 to −0.31) (p < 0.001) Z-SOS −0.55 (−0.89 to −0.21) (p < 0.01) Model2 (F): Z-BUA −0.56 (−0.85 to −0.28) (p < 0.01) Z-SOS −0.47 (−0.78 to −0.16) (p < 0.01) Model3 (F): Z-BUA −0.54 (−0.90 to −0.19) (p < 0.01) Z-SOS −0.55 (−0.95 to −0.15) (p < 0.01)	
										Model1 (M): Z-BUA −0.26 (−0.77 to 0.25) Z-SOS −0.50 (−1.03 to 0.03) Model2 (M): Z-BUA 0.03 (−0.49 to 0.54) Z-SOS −0.13 (−0.75 to 0.49) Model3 (M): Z-BUA 0.15 (−0.41 to 0.72) Z-SOS 0.23 (−0.41 to 0.87)	
Renn et al. [83] (Taiwan)	Cross-sectional Clinical (2003-2004) Na	DSM-IV	QUS-II: calcaneal Ultrasonometer (Metra Biosystems, Mountain View, CA, USA)	BUA T-score	N = 965 F 342 (35%) Nr	F = 46.8 ± 11.2 (20–78) M = 47.6 ± 15.9 (21–95)	N = 405 F 222 (55%) Nr	F = 54.2 ± 16.0 (20–88) M = 60.0 ± 18.2 (20–89)	Matched: Nr Ratio: Nr Adjusted: Na	BUA (Community vs. SCHz) = (B = 13.069, SE = 4.096, p < 0.01)	5/13
Rey-Sánchez et al. [82] (Spain)	Nr Clinical (Nr) Na	DSM-IV CIE-10	QUS: ultrasound device model DBM, Sonic 1200	Ad-SoS	N = 73 F 25 (34.2%) postmenopausal	F = 59.8 ± 17.0 (Nr) M = 61.9 ± 12.9 (Nr)	N = 73 F 25 (34.2%) postmenopausal	F = 60.4 ± 17.1 (Nr) M = 61.2 ± 13.1 (Nr)	Matched for: age, weight, height, gonadal status Ratio: 1:1 Adjusted: Na	SCHz (F) = 2003 ± 86 HC (F) = 2050 ± 66 p < 0.05 SCHz (M) = 2068 ± 59 HC (M) = 2037 ± 69 p < 0.05	5/13

*SCHz* Schizophrenia, *OR* odds ratio, *SD* standard division, *CI* confidence interval, *SCID-I* Research Version of the Structured Clinical Interview for Diagnostic and Statistical Manual of Mental Disorders, *DSM* Diagnostic and Statistical Manual of Mental Disorders, *QUS* Quantitative Ultrasound, *BUA* Broadband Ultrasound Attenuation, *SOS* Speed Of Sound, *Ad-SoS* amplitude-dependent speed of sound, *Z-score* calculated with the formula Z _ (S-AG)/SDAG, where S is the subject’s result, AG is the age- and sex-matched mean value in our study population, and SDAG is the standard deviation (SD) around that mean; *T-score* The t-score was calculated using machine BUA value and adjusted as: (BUA individual – 92.72)/13.36 for the male, and (BUA individual – 87.90)/10.68 for the female, *M* male, *F* female, *Nr* not reported, *Ns* not significant, *Na* not applicable.

**Table 4:** Characteristics and summary of the included studies investigating schizophrenia and bone turnover markers.

Citation (Country)	Study design, setting (years)	Exposure diagnostic criteria	Outcome assessments (Method)		Study population characteristics		BTMs assessed	Results		Matching Ratio (sample/case: control) Adjusting confounders	NIH score
				Subjects	N=total Female N (% of total) Menopause status	Mean age ± SD (range)		Mean ± SD/IQR	P values		
Altindaǧ et al. 2009 [49] (Turkey)	Nr Clinical FU:6 months after they done (July-Dec 2008)	DSM-IV	BTMs (ELISA method)	SCHz	N=30 F (100%) Premenopausal	42.8 ± 5.3 (23-52)	ALP	139.8 ± 27.0	p>0.05	Matched for: sex Ratio: Nr Adjusted: Nr	4/13
				HC	N=40 F (100%) Nr	36.9 ± 8.4 (32-51)		144.6 ± 19.1			
Bergemann et al. 2008 [60] (Germany)	Nr Clinical (Nr)	DSM-IV ICD-10	BTMs (high-performance liquid Chromatography luminescence immunoassay)	SCHz	N= 72 F (100%) Premenopausal	33.8 ± 6.5 (20.5-45.3)	OC PYD DPD	5.4 ± 2.3 59.0 ± 36.3 6.5 ± 3.3	p<0.001 p<0.001 p=0.001	Matched for: age, sex Ratio: Nr Adjusted: Nr	5/13
				HC	N=71 F (100%) Nr	33.7 ± 6.3 (20.9-45.9)	OC PYD DPD	2.5 ± 1.9 40.0 ± 23.3 4.7 ± 3.3			
Chiang et al. 2020 [62] (Taiwan)	Cross-sectional Clinical (Nr)	DSM-IV	BTMs (Nr)	SCHz	N= 47 F 20 (42.55%) Nr	41.9 ± 10.0 (Nr)	ALKP OC	70.60 ± 18.57 18.31 ± 7.39	p=0.035 p=0.096	Matched: Nr Ratio: Nr Adjusted: Na	7/13
				HC	N= 39 F 28 (71.79%) Nr	39.46 ± 8.1 (18-65)	ALKP OC	49.95 ± 15.20 14.62 ± 5.32			
Doknic et al. 2011 [64] (Serbia)	Prospective, cross-sectional Clinical (Nr)	DSM-IV	BTMs (Electrochemiluminescence immunoassay ECLIA)	SCHz	N= 26 F 14 (53.84%) Nr	31.3 ± 1.3 (Nr)	OC CTx	18.0 ± 1.0 444.4 ± 39.6	p=0.300 p=0.023	Matched for: age, sex, BMI, education Ratio: Nr Adjusted: Na	5/13
				HC	N=35 F 24 (68.57%) Nr	32.2 ± 1.4 (Nr)	OC CTx	16.1 ± 1.4 323.8 ± 33.6			
Du et al. 2020 [65] (China)	Nr Clinical (Nr)	DSM-5 SCID-P	BTMs (sandwich enzyme-linked immunosorbent assay, ELISA)	SCHz	N = 79 M 100% Na	Mean age = 46 (36-57)	BAP TRACP-5b	IQR= 22.8-34 IQR= 3.8-5.8	p=0.282 p=0.002	Matched for: sex Rati: Nr Adjusted: Na	6/13
				HC	N = 56 M 100% Na	Mean age = 44 (Nr)	BAP TRACP-5b	IQR= 24.43-35.25 IQR= 2.83-4.98			
Lin et al. 2019 [72] (Taiwan)	Prospective^b^ Longitudinal Clinical (Nr)	DSM-IV	BTMs (Nr)	SCHz	N = 111 F 46 (41.44%) Nr		ALP	66.0 ± 17.9 (Non-clozapine) 69.4 ± 23.2 (Clozapine)		Matched for: age Ratio: Nr Adjusted: Na	8/14
				HC	N = 44 F 31 (70.5%) Nr	40.2 ± 8.6		50.6 ± 14.4	p<0.001^a^		
Okita et al. 2014 [84] (Japan)	Nr Clinical (Nr)	DSM-IV-TR	BTMs (commercial enzyme immunoassay)	SCHz	N= 167 F 85 (50.90%) Premenopausal	36.7 ± 9.5 (Nr)	TRACP-5b	296.2 ± 111.5 (M) 211.9 ± 86.2 (F)	Ns p<0.01	Matched: Nr Ratio: Nr Adjusted: Na	4/13
				HC	N= 60 F 36 (60%) Nr	37.0 ± 9.7 (Nr)		322.1 ± 79.7 (M) 278.4 ± 118.2 (F)			
Rey-Sánchez et al. 2009 [82] (Spain)	Nr Clinical (Nr)	DSM-IV CIE-10	BTMs (Hitachi automated analyzer with a-naphthyl substrate, using a reagent from Boehringer Laboratories)	SCHz	N=73 F 25 (34.2%) Postmenopausal	F = 59.8 ± 17.0 (Nr) M = 61.9 ± 12.9 (Nr)	ALPH	1.62 ± 0.73 (M) 3.2 ± 0.92 (F)	Ns p<0.0001	Matched for: age, weight, height, gonadal status Ratio: 1:1 Adjusted: Na	5/13
							TRAP	53 ± 11 (M) 72 ± 12 (F)	Ns p<0.0001		
				HC	N=73 F 25 (34.2%) postmenopausal	F= 60.4 ± 17.1 (Nr) M= 61.2 ± 13.1 (Nr)	ALPH	1.51 ± 0.60 (M) 1.60 ± 0.62 (F)			
							TRAP	48 ± 7 (M) 58 ± 8 (F)			
Zhang et al. 2016 [85] (China)	Nr Clinical (Nr)	DSM-IV	BMTs (An automatic electrochemical luminescence immunity analyzer)	SCHz	N = 116 F 59 (50.86%) Nr	Mean age= 28.6 (18-40)	OC	25.37 ± 10.46 (first-episode) 24.05 ± 9.81 (antipsychotic)		Matched: Nr Ratio: Nr Adjusted: Na	5/13
							Β-CTX	0.52 ± 0.28 (first-episode) 0.69 ± 0.24 (antipsychotic)			
				HC	N = 71 F 39 (54.92%) Nr	30.2 ± 5.0 (Nr)	OC Β-CTX	15.17 ± 4.64 0.32 ± 0.12	p<0.001^a^ p<0.001^a^t		

*SCHz* Schizophrenia, *OR* odds ratio, *SD* standard division, *SCID-I* Research Version of the Structured Clinical Interview for Diagnostic and Statistical Manual of Mental Disorders, *DSM* Diagnostic and Statistical Manual of Mental Disorders, *ICD* International Classification of Diseases, *BTMs* Bone Turnover Markers, *OC* serum Osteocalcin, *ALP* ALKP, *AlPH* Alkaline Phosphate, *PYD* Total Pyridinoline, *DPD* Total Deoxypyridinoline, *CTx* C-terminal telopeptide of type I collagen, *CTX* crosslaps, *BAP* Bone Alkaline Phosphate, *TRACP-5b* Tartrate resistant acid phosphatase isoform 5b, *TRAP* Tartrate-resistant acid phosphatase, *B-CTX* Beta CrossLaps, *Nr* not reported, *Ns* Not significant, *F* female, *M* male, *HC* healthy control.

^a^Both sup-groups of schizophrenia compared with one control group, thus, one P value reported.

^b^Baseline data reported.

### Narrative synthesis

The characteristics of the included studies and summary of findings according to each outcome are presented in Tables 1–4 and described in the following sections, respectively.

#### Schizophrenia and BMD

##### Study characteristics

There were 17 studies published between 1997 and 2021 that examined associations between schizophrenia and BMD [49, 50, 60–74]. The sample sizes ranged between 14 [70] to 229 [67] adults with schizophrenia. Of the 17 studies, 12 were conducted in Asia (70.6%; with 29.4% in China), three in Europe (17.6%) and two in North America (11.7%).

Only one study was conducted within a population-based setting (Manitoba Bone Density Program database) [61], with all other studies conducted within clinical settings. Among these studies, 15 [49, 50, 60, 62–68, 70–74] compared BMD between people with schizophrenia and a control group, one study [69] used reference data for age- and race-matched healthy males, while another study [61] compared the percentage of schizophrenia among people with low BMD and normal BMD. Seven studies [60, 61, 64, 68, 69, 71, 73] used an age- and sex-matched control group and other studies used only an age-matched [72] or sex-matched [49, 65] control group. The remaining studies used an aged- and sex-similar [70, 74] or unmatched control group [50, 62, 63, 66, 67].

Two prospective studies [72, 73] measured BMD before and after treatment with antipsychotic medication, of which we only included the baseline data (pre-treatment). Eight studies [62–64, 66–68, 70, 74] investigating BMD were cross-sectional, reporting a single measurement of BMD. Eleven studies [50, 62–64, 66, 67, 70–74] included both sexes, while three studies [65, 68, 69] included men only and the remaining [49, 60, 61] women only. Three studies [49, 50, 60] out of 17 included premenopausal women, and two studies [61, 67] examined postmenopausal women, while the menstruation status was not reported for the remaining studies.

Of the 17 included studies, 12 used DSM-IV/5 alone or combined with other diagnostic criteria for identifying people with schizophrenia [49, 60, 62, 64–67, 69–72, 74], two used ICD [61, 73], one used the SCID [63] and two studies recruited patients with an existing diagnosis of schizophrenia [50, 68].

Most of the included studies that examined BMD used DXA as the ascertainment method; two studies used QUS [63, 74] and another QCT [65]. Two studies [63, 74] reported that QUS values were transformed to ascertain BMD, according to a previously published method (see Colling et al. [75]). BMD sites varied across studies including: the lumbar spine (n = 14) [49, 50, 60–62, 64–68, 70–73], femoral neck (n = 9) [49, 60, 61, 64, 66–68, 70, 71], trochanter (n = 5) [61, 66–68, 71], Ward’s triangle (n = 3) [67, 68, 71], total hip (n = 2) [61, 71] and distal radius (n = 1) [69]. Two studies used QUS and subsequently reported bone density of the right heel [63, 74]. The highest number of sites assessed was reported by Liang et al. [71], measuring multiple sites, including lumbar spine: L1, L2, L3, L4, and L1-4, hip: femoral neck, trochanter, Ward’s triangle, total hip, and radius: one-third distal, ultra-distal, middle distal, and total radius ulna [71]. Thirteen studies [49, 50, 60, 62, 64–69, 71, 73, 74] reported BMD in g/cm^2^, while ten studies [49, 60–63, 66, 67, 69, 70, 72] reported T-scores and Z-scores were reported by five studies [50, 62, 64, 69, 72].

##### Findings

Among the 16 studies using a control group (including one study [69] using reference data as a comparator), 12 (75.0%) found significantly lower BMD in the schizophrenia group compared with a control group in at least one region or at least one patient subgroup [49, 50, 63, 65–72, 74]. Furthermore, in the only case-control study which investigated the prevalence of schizophrenia among adults with low BMD, Bolton et al. reported a schizophrenia diagnosis was associated with increased odds of osteoporotic BMD [adjusted odds ratio (AOR) 1.98, 95% CI 1.0, 3.77] [61]. In addition, sex differences were observed in several studies [50, 66, 67]. In a polish study assessing BMD at L2-L4, the association between schizophrenia and low BMD was observed among women only [50]. Similarly, Jung et al. [66] reported lower BMD at the lumbar spine, femoral neck, Ward’s triangle and trochanter for the total group and females; however, no relationship was detected among the males at any BMD sites. In 2011, Jung et al. reported significantly lower BMD at the lumbar spine, femoral neck, and trochanter for women with schizophrenia; in the male group, significantly lower BMD was recorded at the femoral neck, trochanter and Ward’s triangle compared to controls [67].

Differential results were observed according to specific sites; Keely et al. [68] measured BMD in male samples and observed significantly lower BMD at the lumbar spine, Ward’s triangle and trochanter in the group with schizophrenia, while no association was observed at the femoral neck. Liang et al. [71] measured BMD in 13 sites and documented a significant difference between people with schizophrenia and controls at L3, femoral neck, trochanter, ultra-distal, middle distal, and total radius ulna.

Only one study stratified the sample of patients with schizophrenia by age [69]; males with schizophrenia (age range=31–78 years) who were categorised into five-year age bands were found to have significantly lower BMD at the distal radius in all ages except 30–34, 35–39, 50-54 age groups, compared to the reference data.

In contrast, four [60, 62, 64, 73] studies did not detect any significant differences in BMD between patients with schizophrenia and controls. Bergemann et al. [60] included 72 females with schizophrenia and compared them with 71 age- and sex-matched controls [60] observing no significant differences in mean BMD T-score at the femoral neck and lumbar spine [60]. Doknic et al. [64] conducted a cross-sectional study comparing the lumbar spine and femoral neck BMD of 26 patients with schizophrenia with 35 age-, sex-, body mass index (BMI)-, and education-matched healthy controls [64], and reported a trend for reduced BMD at the lumbar spine for patients with schizophrenia. However, no difference was observed for femoral neck BMD between patients and controls [64]. A prospective cohort study compared L1-L4 BMD of 163 patients with schizophrenia to 90 matched controls (age-, sex-, BMI-, marital status-, and years of education-matched) and observed no significant differences in baseline BMD between controls and patients (all p > 0.05) [73]. A recent cross-sectional study conducted in Taiwan explored L2-L4 BMD in 47 patients with schizophrenia and 39 controls and found BMD was similar in the two groups [62].

Overall, a total of 110 analyses were extracted from 17 studies; from which, 98 analyses measured BMD. Of 98 analyses, 65.3% indicated significantly lower BMD in people with schizophrenia, while 34.7% did not observe significant results for an association between schizophrenia and BMD. In addition, out of 64 analyses reported significant association, 53.1% reported significant lower BMD in both sexes, with 31.3% in women and 15.6% in men. Taken together, these results suggest that people with schizophrenia have significantly lower BMD than controls.

#### Schizophrenia and fracture

##### Study characteristics

Published between 2004 and 2022, there were eight studies that investigated fracture risk in individuals with schizophrenia compared to controls without schizophrenia [27, 67, 76–81]. Sample sizes ranged from 46 [79] to 30,335 [27] adults with schizophrenia. Three studies were conducted in the USA (37.5%), two in Europe (25.0%) and three in Asia (37.5%).

Six studies used population-based data [27, 76–78, 80, 81]; all population-based studies except one [78] had age- and sex-matched controls and/or adjusted for age and sex. Among the population-based studies, two studies [76, 77] used a case-control design, including 32,133 cases with a fracture and 77,178 controls without a fracture [76, 77]. The two remaining studies used clinical data [67, 79], and matched for age and sex. All included studies comprised both sexes in their study population, except for one study [79] that focused on women only.

Six studies [27, 76, 78–81] used ICD and two studies [67, 77] used DSM to identify people with schizophrenia. Across the studies investigating fracture, six studies used ICD diagnostic criteria to identify fractures [27, 76–78, 80, 81], one used confirmed radiograph reports to identify fractures [67], and the other recorded bone fractures [79]. Hip fracture was the most commonly investigated fracture in people with schizophrenia [27, 76–78, 80, 81], with femoral neck fractures the second most investigated site [76]. In addition, two cohort studies from Canada and Taiwan examined the association between schizophrenia and major osteoporotic fracture (MOF), including hip, spine (clinical), wrist, humerus and forearm [27, 78].

##### Findings

Four studies reported a relationship between schizophrenia and fracture [67, 77, 79, 81]. In a retrospective study, Bishop et al. [79] noted the rate of fracture in 46 women with schizophrenia was significantly higher than that in the 46 age- and sex-matched controls [12/46 (26.1%) vs. 1/46 (2.2%), p < 0.001] [79]. In a cross-sectional study conducted by Jung et al. [67], 229 inpatients with schizophrenia reported a significantly higher lifetime prevalence of fracture compared to 125 healthy controls [55/229 (24.0%) vs. 7/125 (5.6%), p = 0.001] [67]. Furthermore, among those with schizophrenia, 16 out of 229 (6.9%) experienced two or more fractures; however, no controls had more than one fracture [67]. A Canadian population-based study, using an administrative database consisting of 15,792 persons with osteoporotic fractures and 47,289 controls (matched for age, sex, ethnicity, and comorbidity), reported that a diagnosis of schizophrenia was more prevalent among the fracture group, compared to the non-fracture group (OR 2.17, 95% CI 1.75, 2.69; p < 0.05) [77]. In a multivariable model, simultaneously adjusted for antipsychotics, a diagnosis of schizophrenia was still significantly associated with increased odds of fracture (adjusted OR 1.61, 95% CI 1.27, 2.04; p < 0.01) [77]. In a recent nationwide population-based cohort study conducted in Taiwan, including 2028 people with schizophrenia and 8112 controls, a higher incidence of new fracture [89 out of 2028 (4.4%) vs. 257 out of 8112 (3.2%) p < 0.01], hip fracture [25 out of 2028 (1.2%) vs. 54 out of 8112 (0.7%) p = 0.01], and vertebral fracture [53 out of 2028 (2.6%) vs. 142 out of 8112 (1.7%) p = 0.01], in the schizophrenia group was reported. However, the incidence of wrist fracture was similar between schizophrenia and control groups.

However, similar findings were not observed in the remaining studies [27, 76, 78, 80], with the authors suggesting that the relationships between schizophrenia and fracture could be explained by antipsychotic medication use in these studies. For example, in a population-based case-control study conducted in the UK, 16,341 cases with fracture and 29,889 adults without fracture as controls [76] were included in a univariate analysis—reporting that hip fracture was associated with schizophrenia (OR 1.73, 95% CI 1.32, 2.28). However, this association was explained by the addition of antipsychotic medication to the model as a potential confounder (OR 1.01, 95% CI 0.72, 1.40; p = 0.971) [76]. A Danish population-based study consisting of 15,431 people with schizophrenia and 3,807,597 individuals from the general population without a diagnosis of schizophrenia reported similar findings [80]. For example, schizophrenia was associated with a significantly higher incidence rate ratio (IRR) of hip fracture (IRR 1.19 95% CI 1.08, 1.13) after adjustment for all covariates except antipsychotic use; after adding antipsychotic use to the model, the association between schizophrenia and hip fracture was no longer significant (IRR 1.00, 95% CI 0.90, 1.11) [80]. In a retrospective population-based cohort conducted in Taiwan using data from 30,335 people with schizophrenia and 121,340 age- and sex-matched controls without a diagnosis of schizophrenia, a higher incidence of MOF was observed in patients with schizophrenia, compared to controls [n = 1,667 (5.5%) vs. 4,257 (3.5%) p < 0.0001]; but there was no significant difference detected in non-MOFs between groups [n = 1,228 (4.1%) vs. 4,886 (4.0%) p = 0.8652] [27]. However, after adjustment for the psychiatric proportion of days covered (PDC), the relationship between schizophrenia and the risk of major osteoporosis fracture was not significant, and the authors suggested that the observed association may be caused by psychotropic medication, not schizophrenia disorder per se [27]. In a Canadian population-based cohort using 68,730 individuals’ data, schizophrenia was significantly associated with MOF [adjusted hazard ratio (aHR) 1.82, 95% CI 1.6, 2.85; p < 0.05] and with hip fracture (aHR 2.34, 95% CI 1.05, 5.21; p < 0.05) [78]. Nevertheless, when medications were analysed with mental disorders in the same model, the association between schizophrenia and MOF (aHR 1.21, 95% CI 0.75, 1.97) and hip fracture (aHR 1.12, 95% CI 0.48, 2.63) were no longer significant [78].

Overall, of eight studies, 50% reported a significant association between schizophrenia and fracture in the first analyses (univariate models or multivariate models without antipsychotics); however, after including antipsychotics in the analyses, the association between schizophrenia and fracture was not sustained. Thus, there is a possibility that the association between schizophrenia and fracture could be due to related medications, with antipsychotics being a recognised and important risk factor for fracture [27, 76, 78, 80].

#### Schizophrenia and bone quality

##### Study characteristics

Three studies examined bone quality in patients with schizophrenia (n = 1086) and controls without schizophrenia (n = 6578) [34, 82, 83], published between 2009 and 2010. The sample size ranged from 48 [34] to 965 [83] adults with schizophrenia. Two studies were conducted in Europe (66.6%) and one in Asia (33.3%).

Two studies used DSM-IV alone or in combination with other diagnostic criteria for selecting samples. One used the Research Version of the Structured Clinical Interview for Diagnostic and Statistical Manual of Mental Disorders (SCID-I) [34]. All included studies examined bone quality by QUS. In two of the studies, skeletal status was indexed by amplitude-dependant speed of sound (Ad-SoS) [82] and BUA [83], while in the third study, skeletal status was expressed by two values of bone quality, BUA and SOS (Z-score) [34].

One of these three included studies had a population-based setting and utilised data from the Health 2000 Survey database [34] with the other two studies being conducted in a clinical setting [82, 83]. Each of the included studies consisted of participants of both sexes. Partti et al. [34] used age- and sex-matched controls for comparing groups [34], while Rey-Sánchez et al. (2010) matched controls by age, sex, height and gonadal status [82].

##### Findings

All included studies found significantly poorer bone quality in the schizophrenia group compared with a control group in at least one value (BUA/SOS) or at least one patient subgroup (male/female). Results of the three studies vary based on confounders such as age [83] and sex [34, 82]. In a study examining Ad-SoS in 73 patients with schizophrenia (34.2% female) and 73 matched controls (34.2% female), women with schizophrenia had significantly lower Ad-SoS (p < 0.05), while men with schizophrenia had significantly higher Ad-SoS (p < 0.05), compared to controls [82]. In a cross-sectional study that recruited 965 patients with schizophrenia aged over 20 years, and 405 community members, it was observed that younger males aged ≤60 years and females aged ≤50 years with schizophrenia had lower BUA compared to controls, respectively [83]. Nevertheless, the same association was not observed in men older than 60 and women older than 50 years, and these groups had higher BUA compared to the control group [83]. In a population-based study using 6241 individuals’ data, Partti et al. reported that people with schizophrenia (n = 48) had significantly lower age- and sex-standardised BUA and SOS compared to the rest of the population [34]. After controlling for the common confounders for osteoporosis, including antipsychotic use, mood stabilising medications and vitamin D, the only significant determinant of low standardised BUA and SOS in women was schizophrenia (Z-BUA = -0.54 95% CI –0.90, –0.19; p = 0.002; Z-SOS = –0.55 95% CI –0.95, –0.15; p = 0.007) [34].

#### Schizophrenia and bone turnover markers

##### Study characteristics

Published between 2008 and 2020, nine studies investigated bone turnover markers in people with schizophrenia (n = 721) and controls without schizophrenia (n = 489) [49, 60, 62, 64, 65, 72, 82, 84, 85]. The sample sizes ranged from 26 [64] to 167 [84] adults with schizophrenia. Six studies were conducted in Asia (66.6%; with two in China 22.2%) and three studies in Europe (33.3%).

All the included studies used DSM-IV/5 alone or combined with other diagnostic criteria for identifying schizophrenia. The examined bone turnover markers varied across the included studies. The bone turnover markers investigated the most frequently in patients with schizophrenia was ALP [49, 62, 72, 82] and OC [60, 62, 64, 85], followed by TRACP-5b [65, 84]. BALP [65], CTx [64], TRAP [82], B-CTX [85], PYD [60] and DPD [60].

All studies were conducted in a clinical setting. Six studies [62, 64, 72, 82, 84, 85] included both sexes, with two studies stratifying the sample by sex in the analyses [82, 84]. Two studies focused only on women [49, 60] and one study only on men [65]. Controls were matched by age [60, 64, 72, 82], sex [60, 64], BMI, education [64], weight [82], height [82], and gonadal status [82].

##### Findings

A total of 19 analyses were conducted across the nine included studies [49, 60, 62, 64, 65, 72, 82, 84, 85]. All of the analyses [60, 62, 64, 65, 72, 82, 85], but two [49, 84] reported at least one higher bone turnover markers in people with schizophrenia compared to controls. Chiang et al. conducted a study with 47 people with schizophrenia and 39 healthy controls and reported significantly higher ALK-P in adults with schizophrenia (p = 0.035) [62]. Similarly, Lin et al., in a study of 111 patients with schizophrenia and 44 healthy controls, observed higher ALP in the schizophrenia group (p < 0.001) [72]. A study in Serbia compared bone turnover markers in 26 people with schizophrenia, and 35 age-, sex-, BMI-, and education-matched controls, and reported higher CTX in patients with schizophrenia (p = 0.023) [64]. In addition, Zhang et al. compared OC and B-CTX in 116 Chinese adults with schizophrenia and compared them with 71 healthy participants—OC and B-CTX were higher in the patient group (p < 0.001) [85].

Moreover, in a study comparing 72 females with schizophrenia with 71 age- and sex-matched controls, significantly higher PYD, DPD, and OC was reported in the patient group (p < 0.001) [60]. For males, one Chinese study observed a significantly increased TRAC-5b in 70 men with schizophrenia compared to 56 healthy controls (p = 0.002) [65]. Sex differences were observed in two studies that examined associations between schizophrenia and bone turnover markers, thus they were stratified by sex. First, in a study of 73 patients with schizophrenia and 73 controls, higher ALPH and TRAP were observed in women with schizophrenia (p < 0.0001), while the same was not observed for men [82]. Second, in a paper by Okita et al., 167 men and women with schizophrenia were compared with 60 controls—lower TRACP-5b was observed in females with schizophrenia (p < 0.01), but not men [84].

Separately, seven of the 19 analyses (36.8%) recorded no significant difference between people with schizophrenia and controls on bone turnover markers. For example, two studies reported no significant difference in OC between the schizophrenia group and the control group [62, 64]. A Turkish study investigated ALP in 30 premenopausal females with schizophrenia and 40 healthy controls and observed no significant difference in ALP between the groups [49]. A study conducted in China with 70 males with schizophrenia and 56 controls reported no significant difference in BAP [65]. The remaining three analyses stratified by sex reported no significant difference in TRACP-5b [84], ALPH and TRAP [82] for men with schizophrenia compared with controls.

In summary, of 19 analyses, 63.2% indicated a significant relationship between schizophrenia and bone turnover markers compared to controls, while 36.8% did not observe any significant association. From 12 significant studies, 50% reported this association in women, 41.7% in both sexes and 8.3% in men. These results suggest that people with schizophrenia have significantly higher bone turnover markers (resorption and formation markers) compared to the healthy controls.

### Meta-analyses

#### BMD

Of 17 studies examining associations between schizophrenia and BMD that were considered for meta-analyses, seven studies were removed due to information deficiency or heterogeneity. Four studies [49, 60, 61, 69] did not report mean BMD for people with schizophrenia or control groups. In addition, three studies used methods other than DXA to ascertain BMD [63, 65, 74] thus, they were excluded. One study [73] reported BMD at the lumbar spine for L1-L4 range, and we considered L4 for this analysis consistent with the previously published systematic reviews [40, 41]. Thus, ten analyses were included in the meta-analyses to examine associations between schizophrenia and BMD.

##### Pooled results for lumbar spine BMD

The pooled lumbar spine BMD for females was calculated from nine [50, 62, 64, 66, 67, 70–73] studies, including 680 participants.

Women with schizophrenia had lower BMD (SMD –0.50, 95% CI –1.05, –0.05) compared to controls, with evidence of heterogeneity (I^2^ = 90.79%, H^2^ = 10.86). The pooled lumbar spine BMD for males that was calculated from ten studies [50, 62, 64, 66–68, 70–73] including 733 participants, and showed that men with schizophrenia have lower BMD (SMD –1.00, 95% CI –1.99, –0.01) compared to controls, but with high heterogeneity (I^2^ = 97.02%, H^2^ = 33.59).

The overall pooled lumbar spine BMD from 19 analyses of included studies [50, 62, 64, 66–68, 70–73] (N = 1413) showed that adults with schizophrenia have lower BMD (SMD –0.74, 95% CI –1.27, –0.20). The overall effect size test (Z = –2.71, p = 0.01) showed that there is a significant association between schizophrenia and lumbar BMD.

The test of group differences by sex was not significant (Q = 0.75, p = 0.39); heterogeneity was observed (I^2^ = 95.09%, H^2^ = 20.36).

**Fig. 3:**
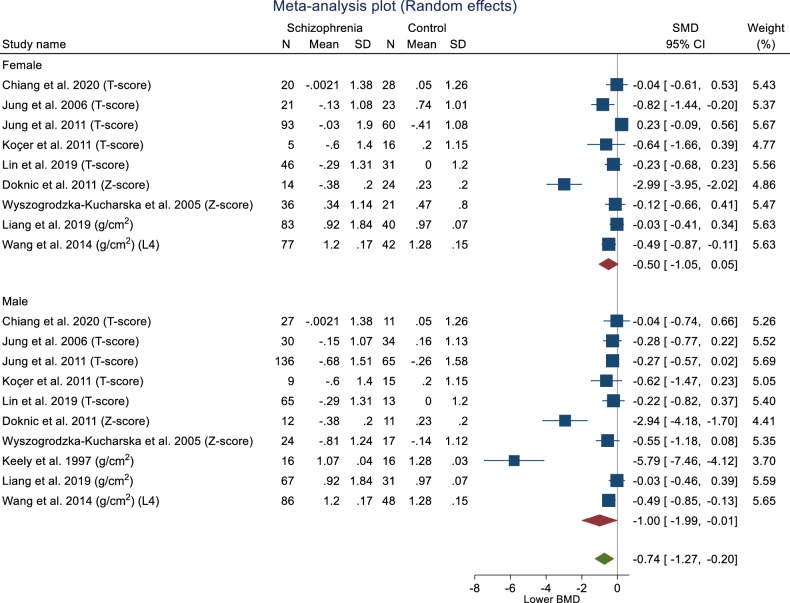
Forest plot for standardised mean differences in BMD at the lumbar spine in people with schizophrenia. SMD < 0 suggests that people with schizophrenia have lower BMD as compared to people without schizophrenia. SMD > 0 suggests that people with schizophrenia have higher BMD as compared to people without schizophrenia. SMD = 0 suggests that BMD is the same for both groups.

**Fig. 4:**
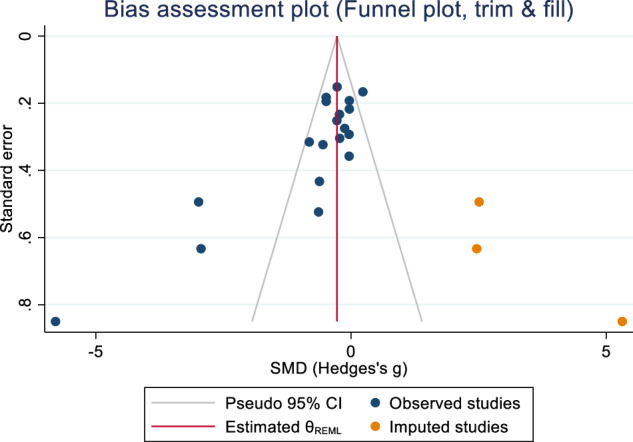
Funnel plot (trim and fill). Publication bias assessment plot with trim and fill for lumbar spine in schizophrenia versus controls.

Publication bias was assessed using Egger’s bias tests (Z = –6.40, p ≤ 0.001) and Begg-Mazumdar: Kendall’s s (Z = –2.52, p = 0.014) and rejected the symmetry of the funnel plot (Fig. 3). Thus, sensitivity analyses were performed to explore the potential sources of the heterogeneity. Subgroup analyses were conducted for publication year and participant size (including samples and controls). Lower heterogeneity was observed when studies [64, 68, 70] with less than 40 in each analysis were removed for women (I^2^ = 54.81%), men (I^2^ = 0.00%) and overall (I^2^ = 37.77%). Meta trim-and-fill reported three hypothetical studies were estimated to be missing. After imputing for those studies, overall bias-adjusted SMD was –0.28 (95% CI –1.07, 0.52) in comparison to the earlier observed SMD –0.74 (95% CI –1.27, –0.20). The bias-adjusted SMD was not significant, indicating that the impact of publication bias was high; in that imputing the missing studies changed the overall result (Fig. 4).

##### Pooled results for femoral neck BMD

The pooled female femoral neck BMD was calculated from five studies [64, 66, 67, 70, 71] with 379 participants. Women with schizophrenia had lower femoral neck BMD (SMD –0.72, 95% CI –1.00, –0.45), with low evidence of heterogeneity (I^2^ = 26.57%, H^2^ = 1.36). The pooled male femoral neck BMD was calculated using six studies [64, 66–68, 70, 71], including 440 participants. Men with schizophrenia had lower femoral neck BMD (SMD –0.89, 95% CI –1.42, –0.36), with evidence of heterogeneity (I^2^ = 80.71%, H^2^ = 5.19). The overall pooled femoral neck BMD using 11 studies [64, 66–68, 70, 71] (n = 819), showed that people with schizophrenia have lower BMD (SMD −0.78, 95% CI −1.03, −0.53).

**Fig. 5:**
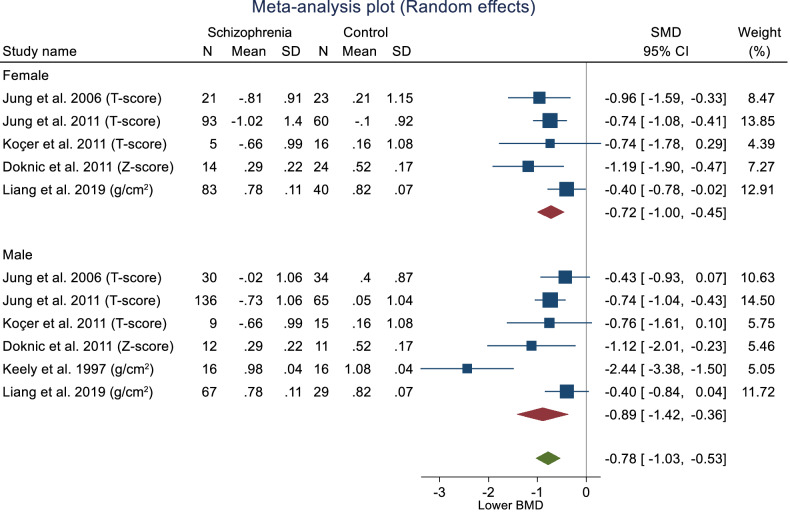
Forest plot for standardised mean differences in BMD at the femoral neck in people with schizophrenia. SMD < 0 suggests that people with schizophrenia have lower BMD as compared to people without schizophrenia. SMD > 0 suggests that people with schizophrenia have higher BMD as compared to people without schizophrenia. SMD = 0 suggests that BMD is the same for both groups.

**Fig. 6:**
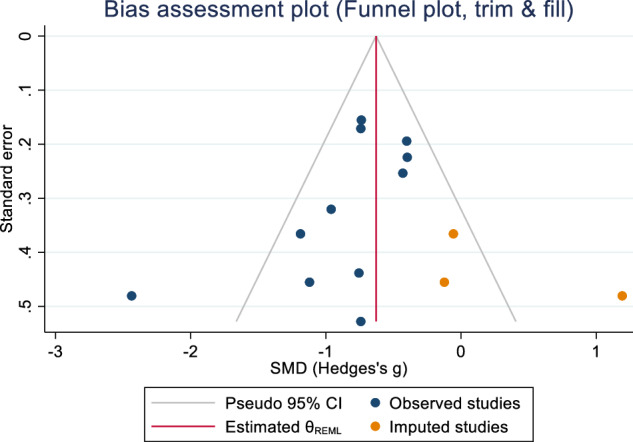
Funnel plot (trim and fill). Publication bias assessment plot with trim and fill for femoral neck in schizophrenia versus controls.

The overall effect size test showed a significant association between schizophrenia and femoral neck BMD (Z = –6.18, p = <0.001). The test of group differences by sex was not significant (Q = 0.30, p = 0.58). However, we observed evidence of heterogeneity (I^2^ = 55.92%, H^2^ = 2.27). Publication bias was assessed using Egger’s bias tests (Z = –2.38, p = 0.017) and Begg-Mazumdar: Kendall’s s (Z = –1.56, p = 0.16) and rejected the symmetry of the funnel plot (Fig. 5). Sensitivity analyses were performed to determine sources of heterogeneity. As per the previous analyses, subgroup analyses were conducted for the publication’s year and participant size (including samples and controls). Lower heterogeneity was observed when one study [68] with a publication year prior to 2000 was removed (I^2^ = 28.25%, 0.00%, 0.00% for female, male and overall, respectively). Meta trim-and-fill reported three hypothetical studies were estimated to be missing. After imputing for those studies, overall bias-adjusted SMD was –0.63 (95% CI –0.97, –0.29) in comparison to the earlier observed SMD -0.78 (95% CI -1.03, -0.54). There is only a slight difference between original and bias-adjusted OR; thus, the impact of publication bias is negligible (Fig. 6).

#### Fracture

Six out of the seven studies were considered for the meta-analyses to examine associations between schizophrenia and fracture. Since two studies [77, 78] were drawn from data from different time points of the same data source (i.e., Manitoba Bone Density Program), it was decided to include the most recent study by Bolton et al. [78].

##### Pooled results for fracture

**Fig. 7:**
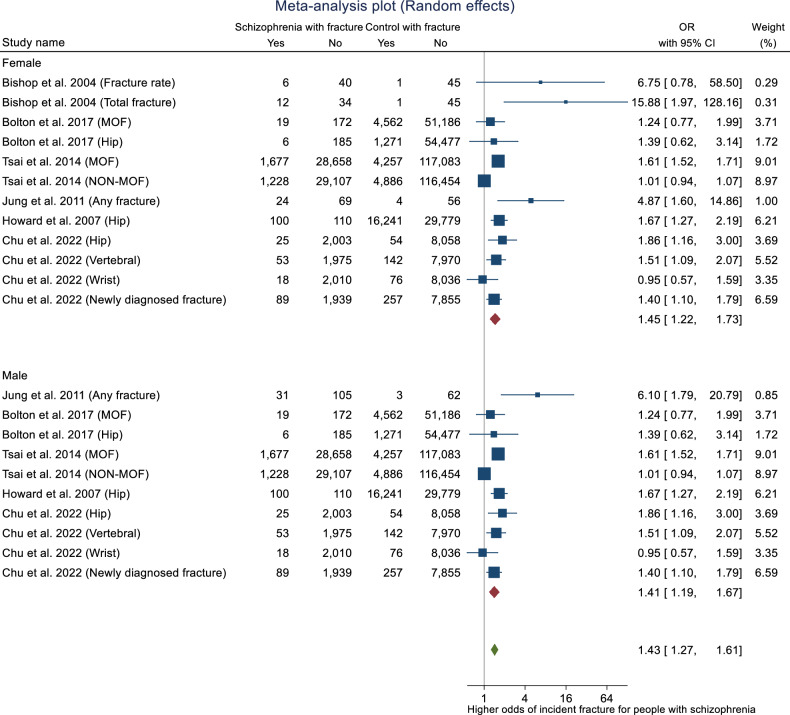
Forest plot for the odds of fracture in people with schizophrenia. OR > 1 suggests that people with schizophrenia have higher odds of fracture as compared to people without schizophrenia. OR < 1 suggests that people with schizophrenia have lower odds of fracture as compared to people without schizophrenia. OR = 1 suggest that the odds of fracture are the same for both groups.

The pooled odds ratio for fracture among females was calculated from seven studies [27, 67, 76, 78–81] with 2,284,378 participants. Women with schizophrenia had a 1.45-fold higher odds of fracture (OR 1.45, 95% CI 1.22, 1.73), with evidence of heterogeneity (I^2^ = 84.71%, H^2^ = 6.54). The pooled odds ratio for fracture among males was calculated from six studies [27, 67, 76, 78, 80, 81] including 1,676,514 participants. Men with schizophrenia had a 1.41-fold higher risk of fracture (OR 1.41, 95% CI 1.19, 1.67), with high heterogeneity (I^2^ = 86.05%, H^2^ = 7.17). The overall pooled odds ratio for fracture, including 3,960,892 participants, showed that people with schizophrenia had a 1.43-fold higher odds of fracture (OR 1.43, 95% CI 1.27, 1.61), with an overall effect size test (Z = 5.88, p = 0.00). The test of group differences by sex did not observe any significant associations (Q = 0.05, p = 0.82). Evidence of heterogeneity was observed (I^2^ = 87.26%, H^2^ = 7.85); thus, sensitivity analyses were conducted to find a source of heterogeneity (Fig. 7). Subgroup analysis for publication year and participant size (including samples and controls), fracture site and methodological quality were conducted, but heterogeneity did not change the results.

**Fig. 8:**
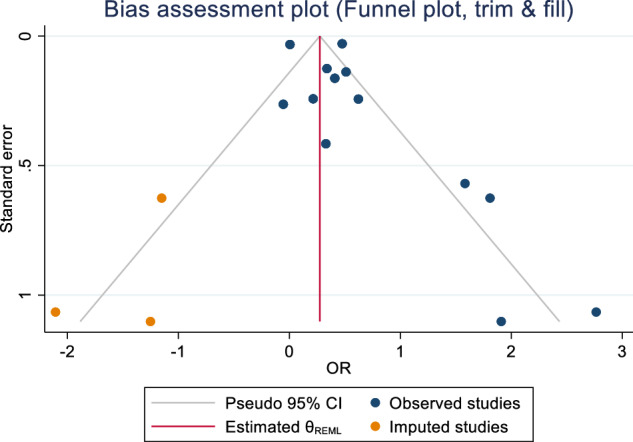
Funnel plot (trim and fill). Publication bias assessment plot with trim and fill for fracture in schizophrenia versus controls. *There is overlap between some studies.

Publication bias was assessed using Egger’s bias tests (Z = 2.95, p = 0.0031) and Begg-Mazumdar: Kendall’s s (Z = 0.48, p = 0.6293) rejected the symmetry of the funnel plot. Meta trim-and-fill reported five hypothetical studies were estimated to be missing. After imputing for those studies, the overall bias-adjusted odds of fracture was 1.32 (95% CI 1.28, 1.35) in comparison to the earlier observed OR 1.43 (95% CI 1.27, 1.61). There is only a slight difference between the original and bias-adjusted OR, with the narrowing of the CIs and likely due to the inclusion of more studies (Fig. 8).

## Discussion

To the author’s knowledge, this is the largest and most comprehensive systematic review conducted to date investigating the associations between schizophrenia and bone fragility, including BMD, fracture, bone quality and bone turnover markers. This systematic review comprised 52,246 individuals with schizophrenia aged between 18-90 years and 4,001,143 controls aged between 18 and 83 years. In aggregate, our results indicate that people with schizophrenia have lower BMD, poorer bone quality and higher rates of bone turnover and fracture than individuals without schizophrenia.

Our findings regarding poor bone health in people with schizophrenia are concerning, given previous studies have indicated that this population experiences more adverse events following a fracture event [36], including longer length of hospitalisation, higher risk of adverse perioperative events and acute post-operative complications [37], and in turn increased healthcare costs [19]. In addition, it has been reported that people with schizophrenia live up to 20 years less than the general population [86]. Thus, it is not unrealistic to suggest that poorer outcomes associated with bone fragility contribute to the higher mortality rate in this population. Given that bone health disorders increase with age, this shortened life span likely reflects an underestimation of the true burden of the disorder.

The underlying reasons for the observed increased risk of bone fragility among people with schizophrenia are complex and likely multifactorial. A diagnosis of schizophrenia is typical between age 10 and 35 years [22], while adolescence plays a vital role in bone health as the amount of BMD gained during this period typically equals the amount lost throughout the reminder of adult life [87]. In addition, several well-known lifestyle risk factors associated with schizophrenia, such as immobility, malnutrition, higher rate of smoking and lower calcium and vitamin D levels, are also associated with bone fragility [28–30, 88, 89]. Furthermore, previous research has shown individuals with schizophrenia may have higher rates of falls and fractures due to antipsychotic use and subsequent dizziness [90–92] and motor coordination difficulty [93]. Furthermore, antipsychotic use could lead to bone loss via antipsychotic-induced hyperprolactinemia as well as other mechanisms such as effects on monoamine bone signalling [31–33, 69, 94]. However, findings are mixed regarding the impact of antipsychotics; while some studies have reported schizophrenia to be independently associated with poorer bone quality [34], increased likelihood of fracture [61] and bone loss [71], antipsychotic use has explained the relationship between schizophrenia and fracture in other studies [27, 77, 78, 80].

Several studies investigated more than one bone outcome. Interestingly, studies investigating bone turnover together with another bone outcome were more likely to report between group differences regarding bone turnover markers than the other measure. For example, three [60, 62, 64] out of six studies (50%) observed a significant association between schizophrenia and at least one bone turnover marker and a non-significant association between schizophrenia and BMD, while only one [49] out of the six studies observed significant association between schizophrenia and BMD but non-significant association between schizophrenia and bone turnover. The two remaining [65, 72] out of the six studies reported a significant association between BMD, bone turnover markers and schizophrenia. These results indicated that bone turnover markers may be more sensitive than BMD in people with schizophrenia, which aligns with the research published in groups from the general population [10]. Another interesting finding was in the paper by Rey-Sánchez et al. [82]. Bone quality in people with schizophrenia was reduced for both sexes with schizophrenia compared to controls, however differences in bone turnover were only observed in women with schizophrenia, not men. Therefore, it is possible that bone turnover may be useful for predicting fracture risk in people with schizophrenia; however, further research to understand sex differences may be needed.

Our meta-analysis showed that people with schizophrenia have lower BMD at the lumbar spine and femoral neck and higher risk of fracture than people without schizophrenia. However, after trim-and-fill adjusting (the method used for adjusting for publication bias), the femur was the only site to remain significant, with a 1.42-fold higher fracture rate observed. These findings could indicate that the femur could be a better site for detecting low BMD among people with schizophrenia. This result is in line with the results of past meta-analyses [19, 39–41] for BMD and the published meta-analysis by Stubbs et al. [26], who also observed a higher fracture rate for people with schizophrenia.

Although our results generally align with previously published meta-analyses, there are some discrepancies. Only one meta-analysis [41] has investigated BMD at different skeletal sites in people with schizophrenia and reported that those with schizophrenia had significantly lower BMD at the lumbar spine (SMD = –0.950) and hip (SMD = –0.534) after adjustment for publication bias. However, after adjusting for publication bias in the current meta-analyses, our results indicated that people with schizophrenia only have significantly lower BMD at the femoral neck (SMD = –0.63). Our results are in contrast with the past meta-analysis by Stubbs et al. [39] in that male sex is a moderator for osteoporosis in people with schizophrenia. The results from our subgroup analyses indicated that lower BMD and higher fracture in this population are independent of sex. These discrepancies could be due to the difference in included studies, and our study included only those with schizophrenia not all psychotic disorders [39, 41]. Elsewhere, one systematic review [19] which included four studies, found antipsychotic use to explain the increased fracture risk in people with schizophrenia. In the current study, we did not investigate the impact of antipsychotics on bone directly. Nevertheless, we should acknowledge that antipsychotic medications are an important risk factor for bone fragility.

Several subgroup analyses were conducted in the meta-analyses of fracture; however, heterogeneity did not change the results. Hence, we should acknowledge there might be some unexpected factors that we did not recognise and the data should be interpreted with caution. These results could be due to the individual variation of studies, different severity of the disease, or some lifestyle factors. Studies providing data on the same lifestyle factors or severity of disease may have the ability to investigate the reason for the heterogeneity of the results.

### Strengths and limitations

In terms of strengths, we provide an up-to-date systematic review and meta-analyses of this growing topic. It extends previous reviews by including bone quality and bone turnover as additional outcomes of interest above and beyond BMD and fracture outcomes that are examined in existing reviews. In addition, we examined more than one BMD site and did not restrict studies to only those published in English. Furthermore, unlike other published systematic reviews and meta-analyses on this topic, the current review focused on schizophrenia only.

In terms of limitations, this study aimed to identify the associations between schizophrenia and bone fragility per se not the role of treatment, which has been associated with poor bone health previously [76, 78, 80]. Secondly, there was considerable methodological heterogeneity observed among included studies. However, we addressed the impact of heterogeneity by conducting stratified and meta-subgroup analyses. For BMD, the heterogeneity was controlled mainly through subgrouping according to the year and number of participants; however, the source of heterogeneity in the fracture meta-analysis was not identifiable. Thirdly, the data extracted from the included studies had heterogeneity and inconsistency in their reporting of results, including for important lifestyle risk factors, which precluded the ability to pool these factors as moderators. Fourthly, Google Translate was used to translate the studies not published in English due to time/resource constraints. Finally, a publication bias was detected by funnel plot and Begg-Mazumdar and Eggers bias tests; nevertheless, trim-and-fill adjustments were conducted for fracture and BMD.

### Conclusion

This systematic review and meta-analysis provides evidence in support of bone fragility in people with schizophrenia. Specifically, people with schizophrenia have lower BMD, particularly at the femoral neck, a higher risk of fracture, poorer bone quality and increased bone turnover. Since osteoporosis is often indetectable before fracture and is associated with multiple detrimental consequences, identifying those at risk of bone fragility is a priority. Further research is needed to evaluate the aetiology of bone fragility in this population and recognise modifiable risk factors such as lifestyle or medications to reduce the potential risk for this patient group. Importantly, there is a need to develop guidelines for preventing risk factors and predicting fracture in people with schizophrenia.

### Supplementary information

PRISMA checklist

Supplementary tables (Search results)

## Data Availability

The original contributions presented in the study are included in the article/supplementary material; further inquiries can be directed to the corresponding author.
