# Supplementary material for: The neglected association between schizophrenia and bone fragility: a systematic review and meta-analyses
Source: Transl Psychiatry. 2024 May 30;14:225. doi: 10.1038/s41398-024-02884-1 (PMC11139985; doi:10.1038/s41398-024-02884-1)
Supplement: Supplementary file 1 — PRISMA checklist [file 41398_2024_2884_MOESM1_ESM.docx]

**Table 1:** Search Results (Embase)

| #1 | 'schizophrenia'/exp |
| --- | --- |
| #2 | 'osteoporosis'/exp |
| #3 | 'metabolic bone disease'/exp |
| #4 | 'fracture'/exp |
| #5 | 'bone'/exp |
| #6 | 'bone density'/exp |
| #7 | 'absorptiometry'/exp |
| #8 | schizophrenia: ti,ab,kw |
| #9 | 'bone mineral density' OR bmd: kw,ab,ti |
| #10 | osteopenia OR 'bone loss': kw,ab,ti |
| #11 | 'dual energy x-ray absorptiometry' OR dxa OR dexa OR densitometry:kw,ab,ti |
| #12 | 'quantitative heel ultrasound': kw,ab,ti |
| #13 | 'bone turnover markers': kw,ab,ti |
| #14 | 'bone quality'/exp |
| #15 | 'bone quality': ab,kw,ti |
| #16 | 'bone fragility'/exp |
| #17 | 'bone fragility': kw,ti,ab |
| #18 | 'bone health'/exp |
| #19 | 'bone health': ab,kw,ti |
| #20 | 'osteopenia'/exp |
| #21 | #1 OR #8 |
| #22 | #2 OR #3 OR #4 OR #5 OR #6 OR #7 OR #9 OR #10 OR #11 OR #12 OR #13 OR #14 OR #15 OR #16 OR #17 OR #18 OR #19 OR #20 |
| #23 | #21 AND #22 |

**Table 2:** Search results (EBSCOhost Medline + APA PsycINFO+ CINAHL)

| 1 | (MH "Schizophrenia+") |
| --- | --- |
| 2 | (MH "Osteoporosis+") |
| 3 | (MH "Bone Diseases, Metabolic+") |
| 4 | (MH "Fractures, Bone+") |
| 5 | (MH "Bone and Bones+") |
| 6 | (MH "Bone Density") |
| 7 | (MH "Absorptiometry, Photon") |
| 8 | AB schizophrenia OR TI schizophrenia |
| 9 | S1 OR S8 |
| 10 | AB (dual-energy x-ray absorptiometry or dexa or dxa or densitometry) OR TI (dual-energy x-ray absorptiometry or dexa or dxa or densitometry) |
| 11 | AB (osteopenia or bone loss) OR TI (osteopenia or bone loss) |
| 12 | AB (bone mineral density or BMD) OR TI (bone mineral density or BMD) |
| 13 | AB (quantitative heel ultrasound or bone turnover markers) OR TI (quantitative heel  ultrasound or bone turnover markers) |
| 14 | AB (bone* N3 (health or quality or fragility) ) OR TI ( bone* N3 (health or quality or fragility) ) |
| 15 | S2 OR S3 OR S4 OR S5 OR S6 OR S7 OR S10 OR S11 OR S12 OR S13 OR S14 |
| 16 | S9 AND S15 |
| 17 | DE "Schizophrenia" OR DE "Acute Schizophrenia" OR DE "Catatonic Schizophrenia" OR DE  "Childhood Schizophrenia" OR DE "Paranoid Schizophrenia" OR DE "Process Schizophrenia" OR DE "Schizoaffective Disorder" OR DE "Schizophrenia (Disorganized Type)" OR DE "Schizophreniform Disorder" OR DE "Undifferentiated Schizophrenia" |
| 18 | DE "Osteoporosis" |
| 19 | "Bones" |
| 20 | DE "Bone Disorders" |
| 21 | TI schizophrenia OR AB schizophrenia |
| 22 | S17 OR S21 |
| 23 | TI ( dual-energy x-ray absorptiometry OR dexa OR dxa OR densitometry ) OR AB ( dual-energy x-ray absorptiometry OR dexa OR dxa OR densitometry ) |
| 24 | TI ( osteopenia or bone loss ) OR AB ( osteopenia or bone loss ) |
| 25 | TI ( bone mineral density or bmd ) OR AB ( bone mineral density or BMD ) |
| 26 | TI ( quantitative heel ultrasound or bone turnover markers ) OR AB ( quantitative heel ultrasound or bone turnover markers ) |
| 27 | TI ( bone* N3 (health OR quality OR fragility) ) OR AB ( bone* N3 (health OR quality OR fragility) ) |
| 28 | S18 OR S19 OR S20 OR S23 OR S24 OR S25 OR S26 OR S27 |
| 29 | S22 AND S28 |
| 30 | (MH "Schizophrenia+") |
| 31 | (MH "Osteoporosis+") |
| 32 | (MH "Bone Diseases, Metabolic+") |
| 33 | (MH "Fractures+") |
| 34 | (MH "Bone and Bones+") |
| 35 | (MH "Bone Density") |
| 36 | (MH "Absorptiometry, Photon") |
| 37 | TI schizophrenia OR AB schizophrenia |
| 38 | S30 OR S37 |
| 39 | TI ( dual-energy x-ray absorptiometry or dexa or dxa or densitometry ) OR AB ( dual-energy x-ray absorptiometry or dexa or dxa or densitometry ) |
| 40 | TI ( osteopenia or bone loss ) OR AB (osteopenia or bone loss ) |
| 41 | TI ( bone mineral density or BMD ) OR AB ( bone mineral density or BMD ) |
| 42 | TI ( quantitative heel ultrasound or bone turnover markers ) OR AB ( quantitative heel ultrasound or bone turnover markers ) |
| 43 | TI (bone* N3 (health or quality or fragility) ) OR AB (bone* N3 (health or quality or fragility) ) |
| 44 | S31 OR S32 OR S33 OR S34 OR S35 OR S36 OR S39 OR S40 OR S41 OR S42 OR S43 |
| 45 | S38 AND S44 |

(MH “Schizophrenia” OR AB “schizophrenia” OR TI “schizophrenia”) and (MH “osteoporosis+” OR MH “bone disease, metabolic+” OR “osteopenia” OR MH “fractures, bone+” OR “bone loss” OR MH “bone and bones+” OR MH “bone density” OR “bone mineral density” OR “BMD” OR “dual energy x-ray absorptiometry” OR “dxa” OR “dexa” OR “densitometry” OR “absorptiometry” OR “quantitative heel unltrasound” OR “bone turnover markers” OR “bone quality” OR “bone fragility” OR “Bone health” OR “bone* N3 (health or quality or fragility)” OR “bone loss” OR
